# Chemical characterization, neuroprotective effect, and *in-silico* evaluation of the petroleum ether extract of three palm tree species against glutamate-induced excitotoxicity in rats

**DOI:** 10.1016/j.heliyon.2024.e39207

**Published:** 2024-10-11

**Authors:** Fatma A. Moharram, Fadila M. Hamed, Elsayed K. El-Sayed, Shimaa K. Mohamed, Asmaa A. Ahmed, Sabah H. Elgayed, Mohammed Abdelrazek, Kuei-Hung Lai, Yara E. Mansour, Mohamed S. Mady, Heba E. Elsayed

**Affiliations:** a Department of Pharmacognosy, Faculty of Pharmacy, Helwan University, Ein Helwan, Cairo, 11795, Egypt; b Department of Pharmacognosy, Faculty of Pharmacy, October 6 University, 6th of October City, Giza, 12585, Egypt; c Department of Pharmacology and Toxicology, Faculty of Pharmacy, Helwan University, Ein Helwan, Cairo, 11795, Egypt; d Department of Pharmacognosy, Faculty of Pharmacy, Cairo University, Cairo, 11562, Egypt; e Department of Cytology and Histology, Faculty of Veterinary Medicine, Cairo University, Egypt; f Graduate Institute of Pharmacognosy, College of Pharmacy, Taipei Medical University, Taipei, 11031, Taiwan; g PhD Program in Clinical Drug Development of Herbal Medicine, College of Pharmacy, Taipei Medical University, Taipei, 11031, Taiwan; h Traditional Herbal Medicine Research Center, Taipei Medical University Hospital, Taipei, 11031, Taiwan; i Pharmaceutical Organic Chemistry Department, Faculty of Pharmacy, Helwan University, Ein Helwan, Cairo, 11795, Egypt

**Keywords:** Arecaceae, Docking, Excitotoxicity, Monosodium glutamate, Neuroprotective, Palm trees

## Abstract

The burden of neurological disorders is growing substantially with limited therapeutic options, urging the consideration and assessment of alternative strategies. In this regard, we aimed to elucidate the phytochemical profile of the petroleum ether extract (PEE) of three palm tree species: *Aiphanes eggersii* Burret, *Carpoxylon macrospermum* H. Wendl. & Drude, and *Jubaeopsis caffra* Becc. (Family Arecaceae), and to evaluate their neuroprotective effect in monosodium glutamate (MSG)-induced excitotoxicity model for the first time. We identified a total of 48, 18, and 45 compounds in *A. eggersii*, *C. macrospermum,* and *J. caffra*, constituting 79.41 %, 60.45 %, and 76.35 % of the total detected compounds, respectively. *A. eggersii* extract was rich in the methyl esters of fatty acids (65.08 %) especially methyl dodecanoate (17.72 %). *C. macrospermum* was exclusively prolific by the triterpene 3*β*-methoxy-d:c-friedo-b’:a'-neogammacer-9(11)-ene (40.36 %), while *J. caffra* was noticeable by hydrocarbons (30.14 %) and lupeol derivatives (19.79 %). The biochemical and histopathological analysis showed that the tested extracts significantly reduced the oxidative stress, especially at the highest tested dose (1000 mg/kg). The extracts also reduced the activity of induced nitric oxide synthetase, Ca^+2^ level, and NR2B subunit expression and attenuated apoptosis and DNA damage. The docking results show that most active natural compounds bind to SOD-1 and NR2B-NMDARs, verifying the credibility of the biological findings. To sum up, the PEE of the three investigated palm tree species possessed a unique blend of lipophilic bioactive constituents that exert promising neuroprotective potential against MSG-induced excitoneurotoxicity. However, further preclinical investigation and pharmaceutical formulation are needed.

## 1 Introduction

Neurological disorders are one of the primary causes of disability and fatalities in middle- and low-income nations [1]. According to the latest analysis from the Global Burden of Disease, Injuries, and Risk Factors [2], about three and a half billion individuals have experienced a nervous system-related condition in 2021. In the last 30 years, the absolute records for disability-adjusted life years and deaths have increased by 15 % and 39 %, respectively. Owing to the global population growth, aging, lifestyle, and environmental risk factors [1]. Among the common lifestyle risk factors is the usage of additives during food processing [3]. Food additives are broadly applied in the modern food industry in minor quantities to improve the organoleptic properties of food [4]. Monosodium glutamate (MSG) is a commonly used flavor and color enhancer that is linked with several health-related problems and neurological toxicity [[5], [6], [7]]. Glutamate is a major excitatory neurotransmitter in the central nervous system. It helps mammals develop memory, learning, and synaptic plasticity. Nevertheless, increased glutamate levels induce excitotoxicity, which has been linked to many neurodegenerative diseases and brain disorders [8]. It opens the post-synaptic N-methyl-D-aspartate receptor (NMDAR), causing Na^+^ and Ca^2+^ influx [9]. Glutamate-induced excitotoxicity results in the over-activation of NMDAR, increased intracellular Ca^2+^ influx, and mitochondrial membrane collapse [[10], [11], [12]]. In addition to, endoplasmic reticulum (ER) stress, DNA damage, reactive oxygen species (ROS), and reactive nitrogen species (RNS) overproduction [[10], [11], [12]]. Accordingly, searching for new therapies targeting excitotoxicity and oxidative stress is a good strategy for potential protection against neurodegenerative diseases.

Natural products are regarded as abundant sources of bioactive compounds with diverse structures and innovative pharmacological activities [13]. The last decade has evidenced a powerful interest in using natural products to promote health outcomes. Natural products offer therapeutic potential for neurodegenerative disorders, *via* neuroprotection and/or neuroregeneration [[14], [15], [16], [17], [18], [19], [20]]. They are considered safer alternatives to pharmacotherapy, with a lesser risk of adverse or withdrawal symptoms [21]. The neuroprotective effect of natural products includes multiple mechanisms such as inhibiting free radicals' production, chelating metals, and exhibiting anti-inflammatory properties [22]. Although many lead compounds are in the preclinical for managing neurodegenerative diseases, the cholinesterase inhibitor galantamine (an isoquinoline alkaloid) is the only natural FDA-approved drug [23]. Natural products’ extracts and mixtures may have beneﬁts over individual natural compounds. As for their multiple targeted approaches, they may present a new avenue for treating excitotoxicity. This underscores the investigation of novel natural extracts with characterized therapeutic mechanisms to promote neuroprotection. Arecaceae is a plant family of 2600 species and 181 genera restricted to tropical and subtropical regions [24]. The majority are well-known as palms, while those with tree-like forms are called palm trees [24]. Species of Arecaceae have great economic and therapeutic importance, as witnessed by coconut products, dates, carnauba wax, and oils [25]. The African palm trees have been used in folk medicine for treating and controlling numerous disorders such as inflammation, epilepsy, headaches, and diarrhea [26,27]. Palm trees are characterized by different phytoconstituents, such as flavonoids, tannins, alkaloids, carotenoids, and lignans [28]. From the biological point of view, palm trees possess antioxidant, cytotoxic, anti-inflammatory, hepatoprotective, and analgesic [28]. The genus Aiphanes comprises twenty-six species, among which *Aiphanes eggersii* Burret is native to Peru and has spiny, solitary pinnate leaves [29]. *Carpoxylon macrospermum* H.Wendl. & Drude, and *Jubaeopsis caffra* Becc. were distinctly belonging to a monotypic genus, with the former being endemic to South Africa. Both are characterized by clustering trunks and alternate, pinnate leaves [29]. On the other side, *J. caffra* is endemic to Vanuatu and distinguished by its pinnately compound leaves [29,30]. Although the phytochemical constituents' heterogenicity and plethora of biological significance were previously reported on palm trees, no previous biological or phytochemical investigations have been reported on these three species.

As per our interest in investigating the biological significance of palm trees, this study aims to explore the neuroprotective potential of the petroleum ether extracts of the three mentioned palm tree species in the MSG-induced excitotoxicity model. In tandem, the phytochemical composition of the tested extracts was unraveled. Ultimately, a molecular docking study was implemented to correlate the variation in the chemical scaffold with the observed activity.

## 2 Material and methods

### 2.1 General experimental materials

Petroleum ether, normal saline, buffered formalin, and phosphate buffer were supplied from El Nasr Pharmaceutical Chemicals Co. (Gesr El Suez, Cairo, Egypt). Monosodium glutamate, ethanol, Ketamine HCl, and hematoxylin-eosin staining solution were obtained from Sigma-Aldrich Inc. (St. Louis, MO, United States).

### 2.2 Plant material and preparation of the petroleum ether extract (PEE)

The leaves of *Aiphanes eggersii* Burret, *Carpoxylon macrospermum* H. Wendl. & Drude, and *Jubaeopsis caffra* Becc. were collected in June 2022 from El Abd Garden, Cairo-Alexandria Desert Road, Egypt (8PXH+64, El Sadat City, Menofia Governorate 6012401), following the local garden's guidelines and Egypt's collection rules. The three species were coded as 01Aeg/2022, 01Cma/2022, and 01Jca/2022, respectively, by Helwan University's Faculty of Pharmacy, Pharmacognosy Department, after being botanically verified by Dr. Trease Labib, Senior Botanist at the Mazhar Botanical Garden in Cairo, Egypt. Shade-dried powdered leaves (1 kg) of *the three species* were separately extracted using a soxhlet apparatus at 40 °C with petroleum ether (3 x 1 L) for 6 h. Extracts were filtered, and the solvent was evaporated under reduced pressure at 40 °C to yield 18, 15, and 14 g of dry extract, respectively.

### 2.3 Phytochemical profile using gas chromatography/mass spectrometry (GC/MS)

The GC/MS phytochemical profile of the three PEEs was carried out using Agilent 7820/5977B Gas Chromatograph/Mass Selective Detector (GC/MSD) (Agilent Technologies, Santa Clara, CA, United States). Extracts were prepared as 10 % stock solution using *n*-hexane, and then 1 μL sample volume was injected using a 7693A automatic liquid sampler (ALS) with a 40:1 split ratio. Components were separated on an Agilent 19091N-136 column (60 m × 0.25 mm ID, and 0.25 μm film thickness) (Agilent Technologies, Santa Clara, CA, United States). Helium was used as carrier gas at a constant flow rate of 5 mL/min, and an injection volume of 1 μL (split ratio of 5:1); Mass spectra were obtained at 70 eV with 0.5 s scan intervals, and fragments range from 50 to 650 Da. Total GC running time was 59 min. The quadrupole and ion-source temperatures were set at 150 and 230 °C, respectively. The initial oven temperature was set at 80 °C (isothermal for 3 min) with a programmed increase of 5 °C/min to 310 °C and a hold time of 10 min. Agilent Mass Hunter Workstation® Software version B.07.00 and PCDL manager B.07.00 (Agilent Technologies, Santa Clara, CA, United States) were used for compound annotations. The percentage of each constituent was calculated from the peak areas delineated in GC spectra. The tentative identification of the detected components was accomplished by comparing the obtained GC/MS spectral data with those recorded in the NIST chemistry webbook library, in addition to previously reported literature.

### 2.4 Animals

Adult *Sprague-Dawley* male rats weighing 180–200 g were purchased from the Egyptian Organization of Biological Products and Vaccines (Helwan, Egypt). Rats were placed in cages with three per cage and sustained at 22 °C ± 2 °C with a 12 h light/dark cycle with unlimited access to food pellet and tap water *ad libitum.* They were housed for seven days as an acclimatization period before the experiment. The Ethical Animal Care and Use Committee, Faculty of Pharmacy, Helwan University, approved the experimental protocol (approval number: A102023). Additionally, it complied with national guidelines for animal care (National Institute for Health and Humanities, Eighth Edition) and the European Community Directive 6/609/EEC.

### 2.5 Experimental design

The three extracts have been tested for their acute toxicity following the protocol stated earlier by our research team [31] and they showed no signs of toxicity or mortality over a wide dose range of up to 5 g/kg. Accordingly, the current experimental design includes sixty-four rats that were assigned into eight groups (n = 8) and administered the vehicle or tested extracts as follows:

Group 1 (control group): rats received the vehicle, normal saline, orally for seven days.

Group 2 (MSG group): rats were given normal saline orally as a vehicle in addition to MSG (i.p., 2 g/kg), 1 h later the dose of the vehicle for seven days.

Groups 3–8 received orally PEE of *A. eggersii, C. macrospermum,* and *J*. *caffra,* respectively (500 and 1000 mg/kg) for seven days, in addition to MSG injection at a dose (2 g/kg/i.p.), 1 h later the doses of the PEE.

The rats were sedated with ketamine (50 mg/kg) and decapitated by cervical dislocation 24 h after the last dose. After separating from the skull, the brains were submerged in ice-cold normal saline. A pair of brains were immersed in 10 % buffered formalin to facilitate histopathological examination. The other six brains were divided into 2 hemispheres; the right hemispheres were homogenized in ice-cold phosphate buffer to yield 10 % (w/v). They were then centrifuged for 10 min at 2000 rpm. The supernatants were stored at − 80 °C for biochemical studies. The other left hemispheres were utilized for reverse transcription polymerase chain reaction (RT-PCR).

### 2.6 Determination of oxidative stress and antioxidant parameters

In brain homogenates, the levels of reduced glutathione (GSH) and nitric oxide (NO) were detected spectrophotometrically adhering to the guidelines included in the kits (BioVision Co, Milpitas, CA95053, United States), Catalog #K464-100, and K 262-200 respectively. As well as the level of malondialdehyde (MDA), superoxide dismutase (SOD), and induced nitric oxide synthetase (iNOS) were determined in brain homogenates using ELISA kits (FineTest Co, Wuhan, 430074, Hubei, China, BioVision Co, Milpitas, CA95053, United States, and Novus Biologicals, Centennial, CO 80112, United States, respectively) following the kit instructions, Catalog #ER1878, E4584-100, NBP2-80257, respectively.

### 2.7 Determination of NR2B subunit expression by reverse transcription polymerase chain reaction (RT-PCR)

The Total RNA was extracted from brain tissue by Direct-zol RNA Miniprep Plus Kit (Catolog#R2072, ZYMO RESEARCH CORP. United States). Then, the SuperScript™ IV One-Step RT-PCR kit was exploited for obtaining cDNA from total RNA (Catalog# 12594100, Thermo Fisher Scientific, Waltham, MA, United States). An Applied Biosystem using software version 3.1 (StepOne™, United States) was implemented to perform real-time PCR amplification and analysis. The used primers are as follows:

**Table:** The primers used in QRT-PCR gene expression of NR2B subunit:

	Forward sequence	Reverse sequence	Gene accession No.
***NMDA-NR2B***	TGGCTATCCTGCAGCTGTTTG	TGGCTGCTCATCACCTCATTC	NM_012574.1
***GAPDH***	GGACTCATGACCACAGTCCAT	CAGGGATGATGTTCTGGAGAG	NM_000194.2

### 2.8 Determination of calcium level

The calcium level in brain homogenate was assessed colorimetrically using a Cell Biolabs, Inc. kit (San Diego, CA, United States, catalog # MET-5121) and observing the instructions provided by the manufacturer.

### 2.9 Determination of 8-Hyroxydeoxyguanosine (8-OHdG) level

8-Hyroxydeoxyguanosine (8-OHdG) was determined in brain homogenate using an ELISA kit (FineTest Co, Wuhan, 430074, Hubei, China, Catalog #EU2548) by applying the instructor's guideline.

### 2.10 Determination of caspase-3 level

The level of Capase-3 was determined in brain homogenate using an ELISA kit (BioVision Co, Milpitas, CA95053, USA, Catalog #E4592-100) by applying the instructor's guideline.

### 2.11 Histopathological examination

The samples of dissected brain tissue were fixed for three days in 10 % neutral buffered formalin. The samples underwent a series of ethanol dehydration steps, followed by xylene clearing, infiltration, and embedding in Paraplast tissue embedding media. Brain tissue sections (4 μm thick) were obtained using a rotatory microtome. For brain tissue examination, the sections were fixed on glass slides. Lastly, tissue slices were examined under a light microscope while being blindly stained with hematoxylin and eosin [32].

### 2.12 Statistical investigation

The results were expressed as mean ± standard error of the mean (SEM). ANOVA and Tukey-Kramer multiple comparison tests were used to determine whether there was any statistical significance between the groups using GraphPad Prism version 8 (GraphPad Software Inc., United States). A significance threshold of p < 0.05 was applied.

### 2.13 Molecular docking study

Using MOE 2014.09, the most effective molecules were created and stored in a molecular database. Protein Data Bank identification codes 5YTO and 5EWJ were used to retrieve the crystal structures of SOD1 and NR2B-NMDARs with inhibitor 964 and QEL, respectively. The structure preparation module of MOE was used to minimize the protein's energy and to protonate it in three dimensions. Before docking, ligand and water molecules were eliminated from the crystal structure. A rigid receptor was used as the docking methodology, and the triangle matcher was used for placement to identify the docking site and dock the database of all the investigated compounds. The rescoring functions London **Δ**G and GBVI/WSA **Δ**G were chosen, with the force field employed for further refinement. The best-scoring conformation for each molecule was determined by minimizing the free binding energy (kcal/mol). The docked conformation of the ligand within the binding site with the greatest docking score has been identified as the most likely binding conformation.

## 3 Results

### 3.1 Phytochemical analysis of the petroleum ether extract (PEE) of *A. eggersii*, *C. macrospermum*, and *J. caffra* using GC/MS

Herein, the GC/MS provides a detailed analysis of the phytochemical profile of the PEE of three palm tree species: *A. eggersii* Burret, *C. macrospermum* H.Wendl. & Drude, and *J. caffra* Becc. (Family Arecaceae) (Supplementary Figs. S1–S3). A total of 48, 18, and 45 compounds have been identified constituting 79.41 %, 60.45 %, and 76.35 % of the total detected compounds, respectively (Table 1, Table 2, Table 3). *A. eggersii* extract was rich in the methyl esters of fatty acids that constitute 65.08 % of the total identified compounds. The extract showed distinct prevalence of methyl dodecanoate (17.72 %), hexadecenoate (8.6 %), and methyl hexadecanoate (6.22 %). The remaining identified percentage was accredited to other phytochemical subclasses such as alcohols (5.37 %), aliphatic hydrocarbons (3.91 %), ketones (3.26 %), aldehydes (1.69 %), and traces of alkenes (0.1 %) (Table 1). On the other hand, the PEE of *C. macrospermum* was exclusively prolific by the triterpene 3*β*-methoxy-d:c-Friedo-b':a'-neogammacer-9(11)-ene (40.36 %). The extract showed diminished ester content (3.7 %) compared to *A. eggersii,* whereas the alcoholic and aliphatic components showed percentages close to those reported in *A. eggersii* (5.52 % and 5.72 %, respectively). Aromatic phthalates were hardly detected (2.08 %), while traces of ethers (0.13 %) and sterols (0.14 %) were noticed. Interestingly, the extract showed the absence of ketones and aldehydes (Table 2). Quite the opposite from the previously mentioned species, the extract of *J. caffra* was noticeable by aliphatic hydrocarbons (30.14 %, Table 3), mainly hentriacontane (23.13 %). Additionally, lupeol-derived triterpenes are considered the second main class of phytochemicals in this extract encompassing 19.79 %. Unlike *C. macrospermum*, ketones (5.81 %) have been detected and dominated by 6,10,14-trimethyl-2-pentadecanone (5.67 %). Esters and alkenes (8.39 %) have been perceived in comparable percentages estimating 8.17 % and 8.39 %, respectively, while aromatics (0.83 %), aldehydes (0.02 %), and alkynes (0.01 %) have been barely detected (Table 3).

**Table 1:** Identified phytochemical components in the petroleum ether extract of *Aiphanes eggersii* Burret leaves.

No.	RT (min)	Compound name	% Area	Reference
1.	12.9902	6-oxabicyclo [3.2.1] octane-7-one	0.16	[1s]
2.	19.8414	1-cyclohexyl-2-hepten-1-one	0.03	
3.	23.0539	4-methyl-cyclohexanol	0.07	[2s]
4.	23.0541	Decanal	0.03	[3s]
5.	24.1813	Methyl heneicosanate	0.24	[4s]
6.	24.4624	Zerumbone	0.17	[5s]
7.	25.8141	Octane	0.10	
8.	26.1772	Tridecanal	0.03	[6s]
9.	26.3016	Methyl nonanoate	0.03	[7s]
10.	26.5512	(2*S,*5*R*)-2-isopropyl-5-methyl hept-6-en-1-ol	0.37	
11.	26.6507	6,10,14-trimethyl-2-pentadecanone	2.90	[8s]
12.	27.0042	Diisobutyl phthalate	3.71	[9s]
13.	27.0485	Ethynylcyclohexanol	0.05	[2s]
14.	28.2062	2,2-dimethyl-3-decene	0.10	[10s]
15.	28.2064	1-hexadecanol	0.04	[11s]
16.	28.2073	4-methyl-2-heptanol	0.10	[12s]
17.	28.3234	Methyl hexadecanoate	6.22	[13s]
18.	29.0342	Methyl decanoate	5.08	[14s]
19.	29.0343	Methyl dodecanoate	17.72	
20.	29.7848	Octanal	0.04	[15s]
21.	29.7855	3,3-dimethyl-hexanal	0.03	[16s]
22.	29.7869	3,3,6-trimethyl-1,5-heptadien-4-ol	0.01	
23.	30.2539	Margaric acid methyl ester	0.72	[17s]
24.	31.4829	Linolelaidic acid, methyl ester	0.86	[18s]
25.	31.5881	Tricyclo[5.1.0.0(2,8)] octan	0.08	
26.	31.6134	Oleic acid, methyl ester	1.20	[19s]
27.	32.1007	Methyl octadecanoate	1.05	[14s]
28.	32.1919	1,14-tetradecanediol	2.05	[20s]
29.	32.3048	Bicyclopentyl	2.41	
30.	32.3085	Hexadecadienoate	3.86	[21s]
31.	32.3088	Hexadecenoate	8.60	
32.	33.1496	Butyl hexadecanoate	0.78	
33.	35.8702	4,8,12,16-tetramethyl heptadecan-4-olide	5.40	
34.	35.9769	2-methylbicyclo [3.2.1] octane	0.24	
35.	36.0775	Butyl oleate	0.35	[9s]
36.	36.5213	Methyl-16-hydroxy-15,15-dimethylhexadecanoate	0.17	
37.	38.7478	Diisooctyl phthalate	2.34	[9s]
38.	39.6465	1,2-cyclohexanedicarboxylic acid, bis(2-ethylhexyl) ester	1.93	
39.	41.2912	3,3-dimethyl hexane	0.09	
40.	41.2917	2,4-dimethyl heptane	0.08	
41.	41.2920	Pentadecane	0.24	[22s]
42.	41.7486	Docosenoate	4.27	[23s]
43.	43.2405	*α*-tocospiro A	2.68	[24s]
44.	44.0519	Eicosane	0.30	[25s]
45.	44.4766	Heptacosanal	1.56	[26s]
46.	45.3169	13-oxapentacosane	0.14	[27s]
47.	46.6318	2,7-dimethyloctane	0.23	[28s, 29s]
48.	47.1336	1,5-dimethoxy-2,4-bis(3- methylphthalidyl)benzol	0.55	[30s]
**Percentage of identified compounds**			**79.4**	
**Percentage of un-identified compounds**			**20.6**	

∗Spectral data was compared to those reported in the NIST chemistry webbook library. S: The references list is available in the supplementary material file.

**Table 2:** Identified phytochemical components in the petroleum ether extract of *Carpoxylon macrospermum* H.Wendl. & Drude leaves.

No.	RT (min)	Compound name	% Area	Reference
1.	21.1490	3-hexen-2,5-diol	0.04	[31s]
2.	24.2789	1-decanol	0.21	[32s]
3.	24.2798	Nonyl methyl ether	0.13	[33s]
4.	26.5600	Cyclopentane	0.26	[21s]
5.	26.5602	*Trans*-bicyclo[4.2.0]octane	0.24	
6.	26.6582	6-methyl-2-heptanone	2.80	[34s]
7.	28.3329	Methyl hexadecanoate	1.33	[13s]
8.	35.8807	4,8,12-trimethyltridecan-4-olide	2.37	[35s]
9.	38.7571	Dicyclohexyl phthalate	0.39	
10.	38.7579	Dioctyl phthalate	1.66	[9s]
11.	43.2474	*α*-tocospiro A	5.27	[24s]
12.	44.0615	Octane	0.29	
13.	46.4786	3*β*-acetoxystigmast-5-en	0.14	[36s]
14.	46.6431	2,7-dimethyl-octane	1.13	[37s]
15.	46.6433	Hexadecane	1.65	[21s]
16.	46.6434	Undecane	2.15	[38s]
17.	47.0928	1,5-dimethoxy-2,4-bis (3- methylphthalidyl) benzol	0.03	[30s]
18.	50.0105	3*β*-methoxy-d:c-friedo-b':a'-neogammacer-9(11)-ene	40.36	[39s]
**Percentage of identified compounds**			**60.45**	
**Percentage of un-identified compounds**			**39.55**	

∗Spectral data was compared to those reported in the NIST chemistry webbook library. S: The references list is available in the supplementary material file.

**Table 3:** Identified phytochemical components in the petroleum ether extract of *Jubaeopsis caffra* Becc. Leaves.

No.	RT (min)	Compound name	% Area	Reference
1.	18.0918	5-methylundecane	0.06	[38s]
2.	23.0787	Nonanal	0.01	[9s]
3.	23.0788	4-methylnonane	0.05	
4.	24.4662	Zerumbone	0.07	[5s]
5.	26.5668	Neophytadiene	1.26	
6.	26.6619	6,10,14-trimethyl-2-pentadecanone	5.67	[8s]
7.	27.0161	Diisobutyl phthalate	0.17	
8.	27.0647	Citronellyl acetate	0.07	[40s]
9.	27.0651	Citronellyl isobutyrate	0.05	
10.	27.5595	*(E)-*6,10-dimethyl-3-undecen-2-one	0.01	
11.	27.5602	3-methyl heptanal	0.01	[41s]
12.	28.2193	Hexa-hydro-farnesol	0.24	[42s]
13.	28.3360	Methyl hexadecanoate	1.02	[13s]
14.	29.7988	Oxalic acid, allyl pentadactyl ester	0.06	[43s]
15.	31.5030	1-undecyne	0.01	[44s]
16.	31.6130	Tricyclo[4.3.1.0(2,5)]decane	0.05	
17.	31.6307	3,6,6-trimethylnopinanol	0.22	[45s]
18.	31.6314	4-hydroxyheptanoic acid lactone	0.10	[46s]
19.	32.1140	Methyl octadecanoate	0.07	[47s]
20.	32.1149	Methyl decanoate	0.02	[14s]
21.	32.3468	2-butylcyclopenanone	0.06	
22.	32.3471	1,2,3,5-tetramethyl cyclohexane	0.29	[9s]
23.	34.2067	Tributyl citrate	0.02	
24.	35.1312	Docosane	0.11	[48s]
25.	35.8862	4,8,12,16-tetramethylheptadecan-4-olide	1.86	[49s]
26.	35.8867	3,4,6-trimethyl-1,5-heptadien-4-ol	0.39	[50s]
27.	36.8753	Terephthalic acid, isobutyl 4-octyl ester	0.25	[51s]
28.	36.8754	Terephthalic acid, isobutyl 4-methylhept-3-yl ester	0.06	[52s]
29.	38.7685	Phthalic acid, di (2-propyl pentyl) ester	4.31	[53s]
30.	39.6600	Diethyl- 2-butyl 1,1-cyclopentane dicarboxylate	0.02	
31.	39.6606	1,2-cyclohexanedicarboxylic acid, di-(2-ethylhexyl) ester	0.09	[54s]
32.	39.8540	Octane	0.01	
33.	41.8506	1,4-benzenedicarboxylic acid, bis(2-ethylhexyl) ester	0.17	[55s]
34.	43.2566	α-tocospiro A	2.34	[24s]
35.	44.0706	Dodecane	0.88	[2s]
36.	44.0708	Nonacosane	2.38	[9s]
37.	45.3850	2,7-dimethyloctane	0.31	[29s]
38.	46.6628	Hentriacontane	23.13	[56s]
39	47.1546	*dl*-*α*-tocopherol	0.25	[57s]
40.	47.8838	3,3,4,4-tetraethylhexane	0.02	[58s]
41.	49.0796	Heptacosane	2.85	[2s]
42.	49.9988	6*S*-2,3,8,8-tetramethyltricyclo [5.2.2.0(1,6)]undec-2-ene	7.13	[59s]
43.	49.9994	Epilupeol; 20(29)-Lupen-3α-ol, methyl ether	17.71	[60s]
44.	50.8597	2-isopropenyl-5-acetyl-4-hydroxy-2,3-dihydro benzofuran	0.41	[61s]
45.	51.6641	Lupeol acetate	2.08	[62s]
**Percentage of identified compounds**			**76.35**	
**Percentage of un-identified compounds**			**24.25**	

∗Spectral data was compared to those reported in the NIST chemistry webbook library. S: The references list is available in the supplementary material file.

### 3.2 Effect of the PEE of *A. eggersii*, *C. macrospermum*, and *J. caffra* on oxidative stress markers

**Fig. 1:**
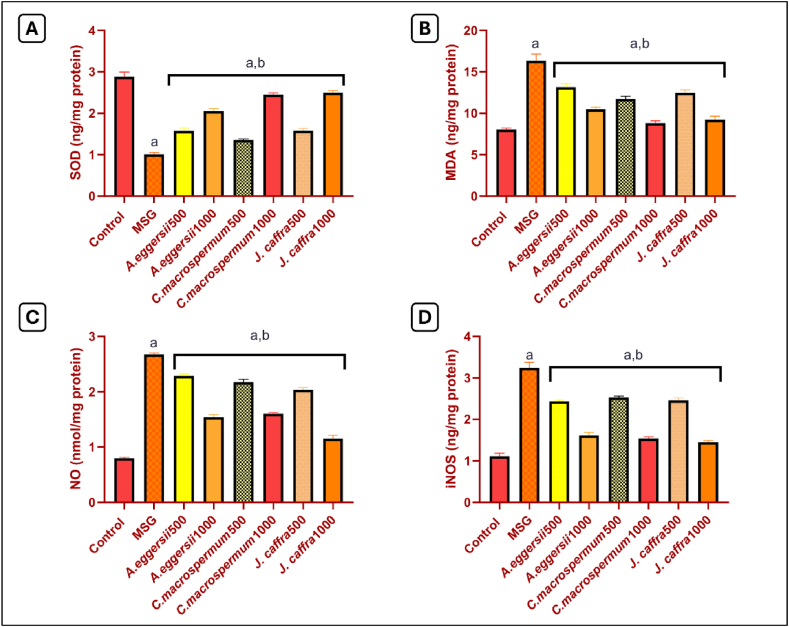
**Effect of petroleum ether extract (PEE) of***A. eggersii***, *C. macrospermum,* and *J. caffra* on (A) SOD, (B) MDA, (C) NO, and (D) iNOS.** Data represented as mean ± SEM of n = 8. a: significant from the control group at *p* < 0.05, b: significant from the MSG-treated group at *p* < 0.05.

Treatment with MSG induced oxidative damage in brain tissue. That is evidenced by the significant decrease in SOD content by 64.9 % and the signiicant (*p* < 0.05) increase in MDA content by 2 folds in MSG-treated rats, compared to the control rats. However, treatment with 500 and 1000 mg/kg of *A. eggersii*, *C. macrospermum,* and *J. caffra* significantly (*p* < 0.05) elevated the content of SOD by 1.6 folds, 2 folds, 1.3 folds, 2.4 folds, 1.6 folds, and 2.5 folds, respectively. In addition, the extracts significantly (*p* < 0.05) decreased the content of MDA by 19.5 %, 35.9 %, 28.3 %, 46 %, 23.6 %, and 43.7 %, respectively, compared to MSG-treated rats (Fig. 1A & B). The MSG-treated group showed a significant (*p* < 0.05) increase in NO content by 3.3 folds compared to the control group. Meanwhile, treatment with *A. eggersii*, *C. macrospermum,* and *J. caffra* in doses of 500 and 1000 mg/kg significantly (*p* < 0.05) decreased NO content by 14.4 %, 42.3 %, 18.7 %, 40.1 %, 23.8 %, and 56.9 %, respectively, compared to MSG-treated rats (Fig. 1C).

### 3.3 Effect of the PEE of *A. eggersii*, *C. macrospermum*, and *J. caffra* on iNOS

MSG-treated rats showed a significant (p < 0.05) 2.9-fold increase in iNOS content compared to the control rats. However, rats treated with 500 and 1000 mg/kg of *A. eggersii*, *C. macrospermum,* and *J. caffra* showed a significant (*p* < 0.05) decrease in iNOS content by 25 %, 50.5 %, 22.1 %, 52.5 %, 24.2 %, and 55.4 % respectively compared to MSG-treated rats (Fig. 1D).

### 3.4 Effect of the PEE of *A. eggersii*, *C. macrospermum*, and *J. caffra* on the relative expression of NR2B subunit

**Fig. 2:**
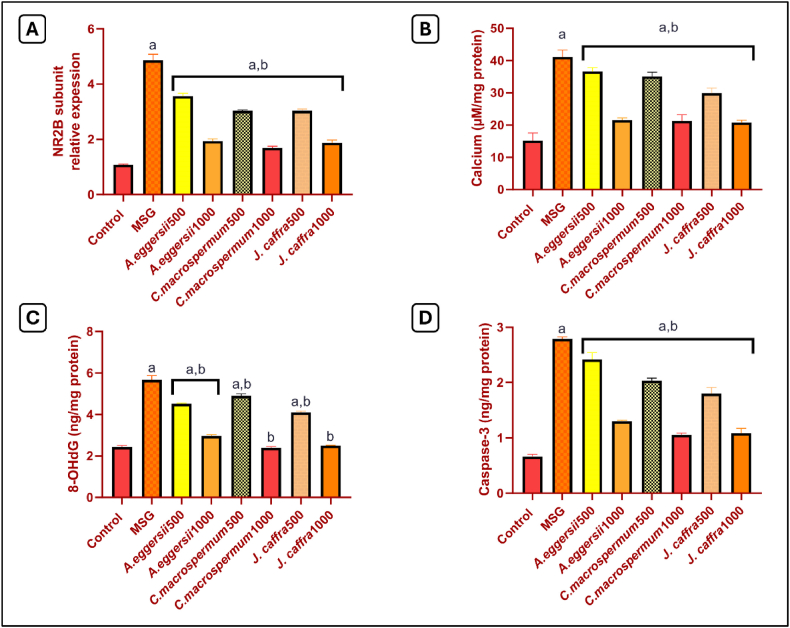
**Effect of the petroleum ether extract (PEE) of***A. eggersii***, *C. macrospermum,* and *J. caffra* on: (A) NR2B subunit, (B) Calcium, (C) 8-OHdG, and (D) Caspase-3.** Data represented as mean ± SEM of n = 8. a: significant from the control group at *p* < 0.05, b: significant from the MSG-treated group at *p* < 0.05.

As represented in Fig. 2A, rats treated with MSG exhibited a significant (*p* < 0.05) increase in NR2B subunit relative expression by 4.5 folds compared to the control rats. However, PEE of *A. eggersii*, *C. macrospermum,* and *J. caffra* in doses (500 and 1000 mg/kg) showed a significant (*p* < 0.05) decrease in NR2B subunit expression by 26.7 %, 60.2 %, 37.6 %, 65.2 %, 37.7 %, and 61.5 %, respectively, compared to MSG-treated rats.

### 3.5 Effect of the PEE of *A. eggersii*, *C. macrospermum*, and *J. caffra* on calcium content

The MSG-treated rats showed a significant (*p* < 0.05) increase in calcium content by 2.7 folds compared to the control rats. However, treatment with 500 and 1000 mg/kg of PEE of *A. eggersii*, *C. macrospermum,* and *J. caffra* caused a significant (*p* < 0.05) decline in calcium content by 10.9 %, 47.8 %, 14.8 %, 48.3 %, 27.3 %, 49.5 %, respectively, compared to the MSG-treated rats (Fig. 2B).

### 3.6 Effect of the PEE of *A. eggersii*, *C. macrospermum*, and *J. caffra* on 8-OHdG content

The MSG-treated rats exhibited a significant (*p* < 0.05) increase in 8-OHdG content by 2.3 folds compared to the control rats. Doses of 500 and 1000 mg/kg of *A. eggersii*, *C. macrospermum,* and *J. caffra* significantly (*p* < 0.05) decreased 8-OHdG by 20.4 %, 47.8 %, 13.7 %, 57.8 %, 27.6 %, and 55.9 % respectively compared to the MSG-treated rats (Fig. 2C).

### 3.7 Effect of the PEE of *A. eggersii*, *C. macrospermum*, and *J. caffra* on caspase-3

Treatment with MSG induced tissue apoptosis, evidenced by the significant (*p* < 0.05) increase in caspase-3 content by 4.2 folds compared to the control rats. However, treatment with 500 and 1000 mg/kg of *A. eggersii*, *C. macrospermum,* and *J. caffra* significantly (*p* < 0.05) decreased caspase-3 content by 13.3 %, 53.5 %, 27.1 %, 62.2 %, 35.5 %, and 61.1 % respectively, compared to the MSG-treated rats (Fig. 2D).

### 3.8 Effect of the PEE of *A. eggersii*, *C. macrospermum*, and *J. caffra* on histopathological examination of brain sections

**Fig. 3:**
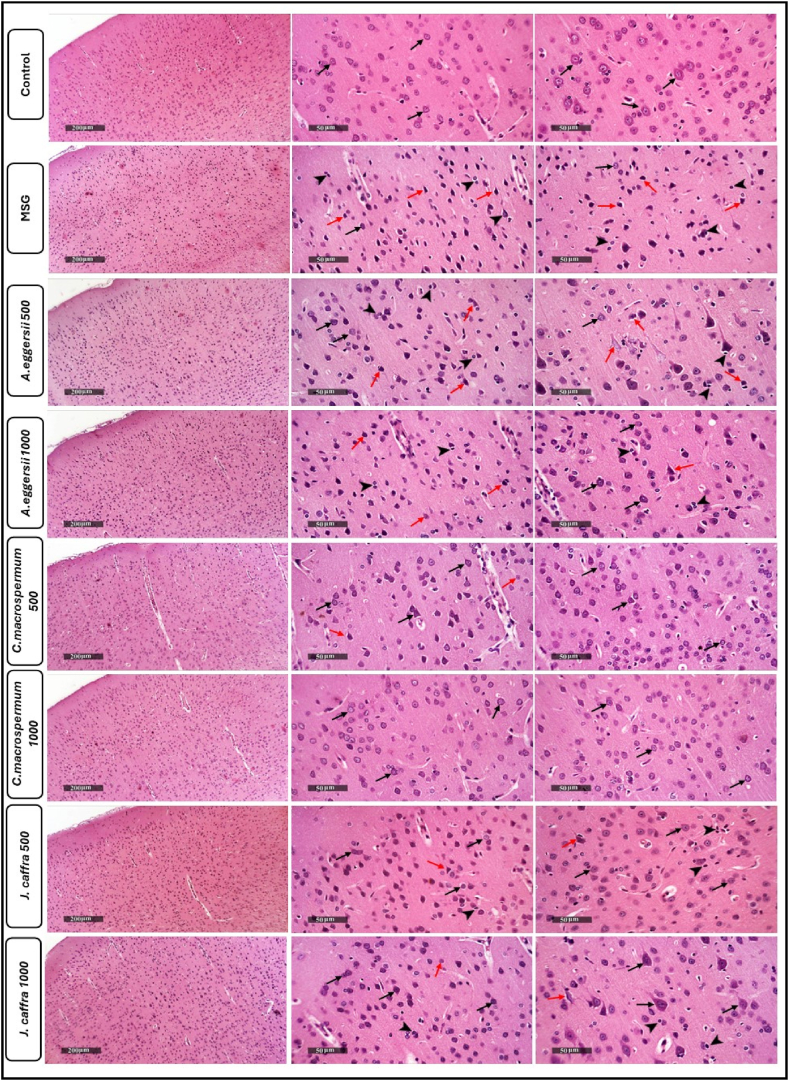
**Effect of the PEE of***A. eggersii***, *C. macrospermum,* and *J. caffra* on histopathological examination of brain sections**. The control group demonstrated intact cortical layers with intact neurons (black arrow) meanwhile, the MSG group showed neuronal degenerative and necrotic damage (red arrow), with minimal intact neuronal cells (black arrow), and microglial cells with neuronophagia (arrowhead). Sections from *A. eggersii* 500 showed neuroprotective efficacy with the presence of intact neurons (black arrow), degenerative changes (red arrow), and reactive glial cell infiltrates (arrowhead). *A. eggersii* 1000 sections showed similar results as *A. eggersii* 500 but with higher neuroprotective effect in the deep cortical layers and increased intact neurons (black arrow). *C. macrospermum* 500 showed mild neuronal degeneration in the outer cortical layers (red arrow) with many intact neurons (black arrow), and intact middle and deep cortical layers (black arrow). *C. macrospermum* 1000 sections demonstrated almost the same as the control group. *J. caffra* 500 showed sporadic neuronal degenerative changes (red arrow), more abundant intact neurons (black arrow), with mild glial cell infiltrates (arrowhead). *J. caffra* 1000 sections showed the same records as dose 500 with higher neuroprotection.

As seen in Fig. 3, the cerebral cortices section from the control group showed intact cerebral cortical layers, intracellular tissue matrix, and whole neurons showing intact cytological details (black arrow). Sections from the MSG group showed neuronal degenerative and necrotic changes (red arrow), minimal records of apparent intact cells (black arrow), and microglial cells with multiple figures of neuronophagia (arrowhead). Sections from *A. eggersii* 500 showed mild neuroprotective efficacy with alternated records of intact neurons (black arrow), abnormal degenerative changes (red arrow), and abnormal reactive glial cell infiltrates (arrowhead). *A. eggersii* 1000 sections showed higher neuroprotective efficacy in deep cortical layers with an increase of apparent intact neurons (black arrow) and persistent records of degenerative changes in outer layers (red arrow) with reactive glial cell infiltrates (arrowhead). *C. macrospermum* 500 sections demonstrated mild neuronal degenerative changes in outer cortical layers (red arrow), many intact neurons (black arrow), and intact middle and deep cortical layers (black arrow). *C. macrospermum* 1000 sections demonstrated almost the same as the control. *J. caffra* 500 samples demonstrated sporadic few neuronal degenerative changes (red arrow), more abundant intact neurons (black arrow), with mild glial cell infiltrates (arrowhead). *J. caffra* 1000 sections demonstrated the same records as dose 500 with higher neuroprotection.

### 3.9 *In-silico* molecular docking evaluation of the major identified compounds

Molecular docking studies were conducted to investigate the mechanical aspects of the major natural products of each extract, methyl dodecanoate, hexadecenoate, methyl hexadecanoate, methyl decanoate, and 4,8,12,16-tetramethyl heptadecan-4-olide in the *A.eggersii; D:C*-Friedo-*B':A′*-neogammacer-9(11)-ene, 3-methoxy-, (3β)- and *α*-tocospiro A in *C. macrospermum* and hentriacontane, Epilupeol and 6,10,14-trimethyl-2-pentadecanone in *J. caffra* in conjunction with the SOD1 and NR2B-NMDARs proteins. This was accomplished with Molecular Operating Environment software (MOE, 2014.10) to probe the complex intermolecular interactions necessary to ascertain the likely binding mode research. Docking the co-crystallized ligand (PDB ID: 946) and the major natural metabolites of the three plant extracts into SOD1 binding sites, the obtained results showed that hentriacontane showed the best binding affinity with a binding score of −8.342 Å in comparison to the co-crystallized Ligand (PDB ID: 946) binding score −6.88 Å (Table 4). α-tocospiro A, 4,8,12,16-tetramethyl heptadecan-4-olide, and 6,10,14-trimethyl-2-pentadecanone showed a binding affinity comparable to the co-crystallized ligand with the binding score (−6.23∼ −6.87 Å).

**Table 4:** Results of molecular docking of the Crystallized Ligand and most identified compounds in conjunction with SOD1 (PDBID: 5YTO).

Docked compounds	PDB ID: 5YTO		
	RMSD (kcal/mol)	Score (Å)	Interacting amino acids
Crystallized ligand (PDB ID:946)	1.01	−6.88	Glu100 = 1.89 Å
			Glu21 = 2.88 Å
			Trp32∼3.62 Å
Methyl hexadecanoate	0.6151	−6.5649	Thr2 = 2.13 Å, ∠ = 157.3°
			Trp32∼4.01 Å
Methyl decanoate	1.4014	−5.5994	Lys23 = 2.24 Å, ∠ = 163.4°
			Trp32∼4.05 Å
Methyl dodecanoate	1.0663	−6.5333	Lys23 = 2.08 Å, ∠ = 156.3°
			Trp32∼4.17 Å
Hexadecenoate	1.6196	−6.3217	Thr2 = 2.04 Å, ∠ = 145.3°
			GLu100 = 2.01 Å, ∠ = 159.6°
			Trp32∼3.85 Å
4,8,12,16-tetramethyl heptadecan-4-olide	1.0994	−6.8764	GLu100C = 3.34 Å, ∠ = 123.3°
			Trp32A∼4.07, 4.17 and 4.20 Å
			Trp32C∼4.01 Å
α-tocospiro A	1.0994	−6.8764	GLu100 = 3.29 Å, ∠ = 126.3°
			Trp32A∼4.01 Å
			Trp32C∼4.25, 4.17 and 4.20 Å
D:C-Friedo-B':A′-neogammacer-9(11)-ene, 3-methoxy-, (3β)-	1.5263	−5.4125	Trp32B∼3.97
			Trp32C∼4.13
6,10,14-trimethyl-2-pentadecanone	1.1380	−6.2394	Glu100 = 2.15 Å, ∠ = 129.5°
			Trp32∼5.33 Å
Hentriacontane	1.5255	−8.3426	Trp32∼3.81 Å
Epilupeol	1.0339	−5.2633	Glu100 = 3.34 Å, ∠ = 156.9°
			Glu21 = 2.88 Å
			Trp32∼4.34 Å

Regarding the NR2B-NMDARs *in-silico* docking study, co-crystallized ligand (PDB ID: QEL) along with the major identified metabolites were docked into NR2B-NMDARs binding sites (PDB code: 5EWJ) and the results (Table 5) showed that hentriacontane achieved good fitting with an efficient binding score of −11.56 Å compared to co-crystallized ligand (PDB ID: QEL) (−10.15 Å). Methyl hexadecanoate, hexadecenoate, methyl dodecanoate, and methyl decanoate docking results showed acceptable binding affinity with docking scores −8.2247, −7.9334, −7.3711 and −6.6813 Å, respectively.

**Table 5:** Results of molecular docking of the crystallized ligand (PDB ID: QEL) and most identified compounds in conjunction with NR2B-NMDARs (PDB ID: 5EWJ).

Docked compounds	PDB ID: 5EWJ		
	RMSD (kcal/mol)	Score (Å)	Interacting amino acids
Crystallized ligand (PDB ID QEL)	1.0005	−10.1571	Gln110 = 2.89 Å, ∠ = 144.3°
			Glu236 = 2.59 Å, ∠ = 150.5°
Methyl hexadecanoate	0.8249	−8.2247	Arg115 = 2.04 Å, ∠ = 157.7°
Methyl decanoate	1.0022	−6.6813	Arg115 = 2.18 Å, ∠ = 120.7°
Methyl dodecanoate	1.2333	−7.3711	Arg115 = 2.12 Å, ∠ = 132.2°
Hexadecenoate	0.4191	−7.9334	Ser132 = 3.41 Å,∠ = 118.3°
			H_2_O = 1.93 Å,∠ = 123.9°
			Tyr175 = 2.3 Å, ∠ = 165°
			Met207 = 2.19 Å, ∠ = 146.6°
4,8,12,16-tetramethyl heptadecan-4-olide	0.8324	−8.2938	Arg115 = 2.12 Å, ∠ = 132.3°
*α*-conspire A	0.8258	−9.3048	Glu106 = 1.77 Å, ∠ = 152.3° (OH-oxaspiro) Arg115 = 1.89 Å (∠ = 137.5°), 2.25 Å (∠ = 128.4°), 1.95 Å (∠ = 157.8°) (O-oxaspiro and O-Acetyl) Phe113 = 3.7 Å (CH_3_-Acetyl)
6,10,14-trimethyl-2-pentadecanone	0.8336	−8.0331	Arg115A = 1.9 Å (∠ = 159.6°), and 2.39 Å (∠ = 138.3°)
Hentriacontane	1.0326	−11.5609	Phe113 = 4.13 Å

## 4 Discussion

Isolated metabolites from natural products are considered a cornerstone of drug discovery. They have been thoroughly investigated to find more potent medications to treat various ailments, including neurodegenerative diseases [14]. Neurodegenerative diseases are triggered by the progressive loss and dysfunction of neurons and neuron-supporting cells in the central nervous system [33,34]. Among the challenges that currently available therapies encounter is that they do not alleviate the whole spectrum of the complicated underlying mechanisms. In addition, the blood-brain barrier (BBB) hampers the efficiency of the applied remedies due to the drug's poor bioavailability [34,35]. Hence, more research is required to investigate other alternatives that could conquer the challenges mentioned above.

Arecaceae species showed a significant impact in the neurological field. For instance, palm oil showed neuroprotective outcomes due to its lipophilic constituents [36]. Also, Marina et al. (2009) have documented the protective potential of the lipophilic components in virgin coconut oil against stroke and degenerative diseases [37]. Furthermore, African palm trees have been used in folk medicine for treating and controlling epilepsy and headaches [26,27]. Given the promising neuroprotective background for some Arecaeae species and the crucial demand for applied research focused on new treatments for neurodegenerative diseases, we aimed to elucidate the lipophilic PEE of three unexplored palm tree species, namely *A.eggersii*, *C.macrospermum* H.Wendl. & Drude, and *J. caffra* Becc. In addition, screen their potential neuroprotective effect in glutamate-induced excitotoxicity *in vivo* model. Petroleum ether is an organic solvent commonly used to extract lipophilic constituents from various plant materials while minimizing the interference of polar components [38,39]. Compound lipophilicity is an important physicochemical property that exhibits an important part in therapeutic drug pharmacokinetics [40]. Additionally, there is a parabolic relationship between lipophilicity and *in vivo* drug brain penetration, where those with reasonable lipophilicity display the highest uptake. Conversely, highly polar compounds are highly soluble in water, rapidly cleared by the kidneys, and have ionizable functional groups that prevent BBB penetration [40]. Consequently, petroleum ether was adopted as an optimal extraction solvent to solubilize lipophilic constituents rather than polar ones. Herein, the PEE of each species was subjected for the first time to GC/MS analysis to unravel their detailed phytochemical profile. The results showed the similarity of some components with those previously reported from other species in the family Arecaceae but they present in our extracts in a unique blend. For instance, *A. eggersii* extract was rich in methyl hexadecenoate, the methyl ester of a long-chain unsaturated fatty acid, which has been reported before in *Chamaerops humilis* [41] and *Livistona australis* [42]. *J. caffra* extract was noticeable by hentriacontane (an alkane hydrocarbon) and lupeol triterpenoids that have been detected in *Chamaerops humili* [41] and *Phoenix paludosa* [43], respectively. On the contrary, *C. macrospermum* was exclusively prolific by the unique triterpene; 3*β*-methoxy-d:c-friedo-b':a'-neogammacer-9(11)-one that has not been reported before in the lipophilic extract of any palm tree species. Interestingly, most of the identified phytoconstituents in the three investigated PEEs were categorized as lipids. Lipids are a broad and common class of molecules that serve a variety of essential biological roles [44]. From a chemical standpoint, they are organic, lipophilic compounds that are water-insoluble but soluble in organic solvents and exhibit remarkable structural diversity, such as variable chain length, a multitude of oxidative, reductive, substitutional, and ring-forming biochemical transformations [45]. They encompass fatty acids and their derivatives, alkanes, sterols, fat-soluble vitamins, glycerides, and others. From the biological significance, lipids are prime elements of the structure and function of the brain [46,47]. They support various physiological functions, including synaptic transmission, protein stabilization, cell signaling and transduction, and cell transport [48,49]. Compared to the other membranes, which contain 40 % lipids, the adult brain's myelin sheaths comprise 70–80 %. Accordingly, examining the neuroprotective significance of the three lipophilic extracts for the first time in glutamate-induced excitotoxicity *in vivo* model was interesting. The neurotransmitter glutamate plays an important role in neuronal functions, initiated by pre-synaptic glutamate release, followed by the post-synaptic activation of the NMDA receptors [50]. The functional NMDAR tetrameric protein comprises two constitutive glycine-binding NR1 subunits and two glutamate-binding NR2 subunits, including NR2A, NR2B, NR2C, and NR2D subunits. Crucially, it has been suggested that this neurotransmitter receptor's unique electrophysiological properties result from combining different NR2 subunits [51,52]. For instance, NMDAR's NR2A versus NR2B ratio changes are linked to neuronal damage [53]. Furthermore, neuroprotection is shown to be provided by the NR2A-dominating combination of NMDAR. During glutamate excitotoxicity, post-synaptic neurons are known to be driven toward apoptosis by NR2B-rich NMDAR [54]. Therefore, modifying the composition of NMDARs to regulate their function without pharmacologically blocking the channel may represent a novel and distinctive cerebral mechanism to prevent neurological disorders caused by NMDAR overactivation [54]. NR2B exhibits a delayed gating kinetics relative to the NR1/NR2A combination, which causes an increase in Ca^2+^ influx and, as a result, quick activation of the downstream signaling pathway [55]. Our study showed an increase in the expression level of NR2B subunit in the MSG group as compared to the treated group, as a refer to neurotoxic insults of increased glutamate level in brain tissue and accompanied by elevated calcium level in brain tissues; however, pretreated groups by PEE of *A. eggersii*, *C. macrospermum*, and *J. caffra* reversed all these results. High levels of glutamate can cause the death of neural cells by generating ROS, RNS, and intercellular Ca^2+^ influx through the NMDA receptor. GSH is a crucial antioxidant in the CNS's cell proliferation, signal transduction, DNA synthesis, and nutrition metabolism. A high glutamate concentration prevents cells from cysteine up-taking, which is involved in GSH synthesis [56,57]. Moreover, increased calcium levels are uptake by the mitochondria matrix, causing an impairment in the electron transport chain and liberation of ROS [58]. The current investigation revealed that the PEE of *A. eggersii*, *C. macrospermum*, and *J. caffra* ameliorate the induced oxidative stress by increasing GSH, decreasing MDA and NO, and decreasing the activity of SOD.

In excitotoxicity, iNOS is upregulated due to increased calcium levels, which activates the nuclear factor kappa-b (NF-κB). This transcription factor is sensitive to calcium and responsible for iNOS gene expression [59]. According to our research, the PEE of *A. eggersii*, *C. macrospermum*, and *J. caffra* reduced iNOS activity compared to the MSG group. Elevated levels of glutamate can cause an increase in mitochondrial respiration and Ca^2+^ uptake with a subsequent escalation in the genotoxic free radicals such as superoxide. The latter can activate caspase-dependent or caspase-independent apoptosis signaling to cause DNA damage and neuronal death [60]. The current study showed attenuation in apoptosis and DNA damage in pretreated groups with the PEE of *A. eggersii*, *C. macrospermum*, and *J. caffra* by decreasing the level of caspase-3 and 8-OHdG. The histological analysis validates each of the previously mentioned findings. We could highlight that the promising observed activity in the three extracts is delineated from the cumulative neuroprotective mechanisms of the individual components. For instance, methyl hexadecanoate, known as methyl palmitate, is a saturated fatty acid methyl ester detected in the three tested PEEs but in dissimilar percentages. A research group by Neumann has reported the ability of methyl hexadecanoate to lessen neuronal cell death and promote neuro-functional outcomes [61].

Lupeol and *dl-α*-tocopherol (i.e. vitamin E) have been detected in the PEE of *J. caffra.* Lupeol is a pentacyclic triterpenoid with several biological functions, including effective antioxidant and anti-inflammatory properties and eminent blood-brain barrier permeability [62]. Also, it exhibited profound neuroprotective properties through several mechanisms. For instance, it reduces NF-*κ*B [63], NO [64], and MDA [65] while increasing the levels of GSH [66]. Additionally, it inhibits mitochondrial ROS and reduces the oxidative burden in Alzheimer-like diseases. On the other side, tocopherol plays a crucial role in maintaining neurological health, as it protects against oxidative stress and suppresses the expression of many genes involved in the development of neurodegeneration [67]. Meanwhile, we cannot neglect the role of constituents detected at a low percentage. Because extracts have multiple, simultaneous target approaches, they may be more advantageous than individual natural compounds. For instance, the sesquiterpenoid zerumbone that is revealed in the PEE of *A. eggersii* and *J. caffra* exhibits an antioxidant effect, anti-apoptosis, anti-inflammatory, and can reduce the onset and progression of neurodegenerative diseases [68].

Another example is the methyl ester of oleic acid detected in *A. eggersii*. Oleic acid is an essential part of membrane phospholipids and is substantially found in myelin sheaths [69]. Several researchers have validated the importance of oleic acid in proper brain development and functioning. As well they correlate the significant regression of its content to the incidence of Alzheimer's disease and major depressive disorder in comparison with the healthy brain [70,71]. This brings us to an interesting fact reported previously by Mett and Müller (2021): medium-chain fatty acid derivatives, such as those detected in our extracts, showed neuroprotective and cognition-enhancing properties in aged Wistar rats [72]. So, to sum up, the three investigated extracts possessed a variable percentage of bioactive lipid derivatives that synchronously showed up with a promising neuroprotective potential. However, among the limitations of the *in vivo* study, is that the extracts' effect was tested against excitotoxicity induced by MSG on the whole brain. Since excitotoxicity is implicated in several neurodegenerative diseases such as Parkinson's and Alzheimer's disease, specific brain regions such as the hippocampus and striatum can be explored. This point will be considered for future work using animal models for Parkinson's and Alzheimer's disease.

**Fig. 4:**
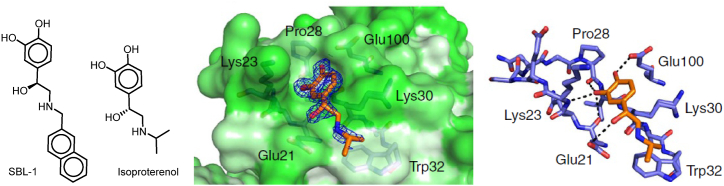
Chemical structure of SBL-1 and isoproterenol and crystal structure of isoproterenol with SOD1 (PDB: 4A7T).

The homodimer metalloenzyme superoxide dismutase 1 (SOD1) is a crucial part of the physical endogenous antioxidant defense mechanism and facilitates the transformation of superoxide anion into hydrogen peroxide. SOD1 mutations have been connected to numerous neurological conditions. These mutations lead to instability, aggregation, and death of motor neurons [73]. The primary approach to prevent the harmful aggregation of SOD1 is to enhance the stability of the SOD1 natural dimer. Conversely, stabilization of the SOD1 dimer has been demonstrated to increase the protein's thermostability, prevent monomerization, and inhibit aggregation [74,75]. SOD1 possesses a remarkably exposed tryptophan residue on the surface of its b-barrel structure. Oxidation *in vivo* can break the Trp32 side chain ring structure, which enhances aggregation and cytotoxicity [[76], [77], [78]]. Prior *in vitro* assay investigated the ability of the isoproterenol compound and its analog SBL-1 to inhibit Trp32 oxidation. The results showed that isoproterenol with small hydrophobic moiety has weak inhibition on Trp32 oxidation and emphasized the importance of π-stacking interactions between the bulky hydrophobic moiety and the indole ring of Trp32 for stabilization of protein-ligand complex and inhibit SOD1 aggregation and neuron survival [79] (Fig. 4).

**Fig. 5:**
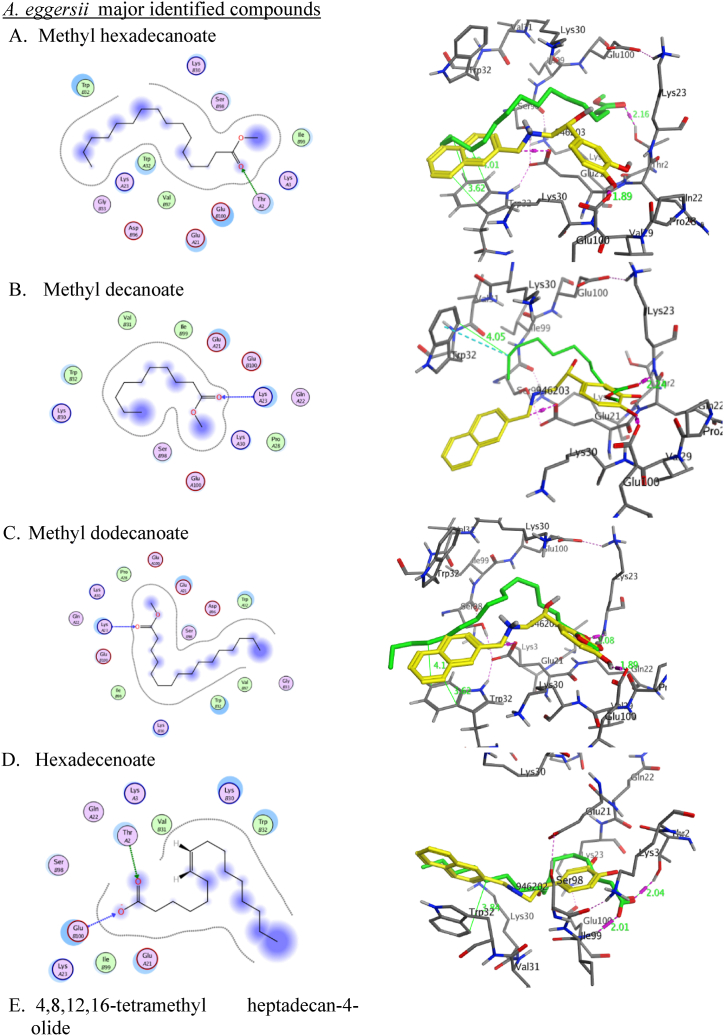

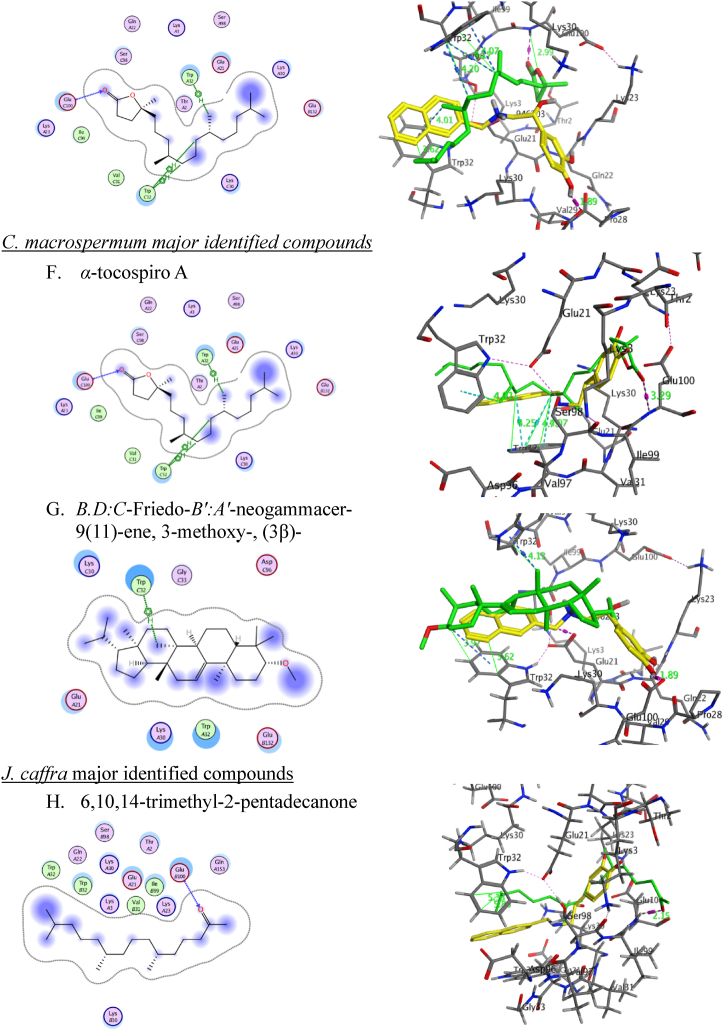

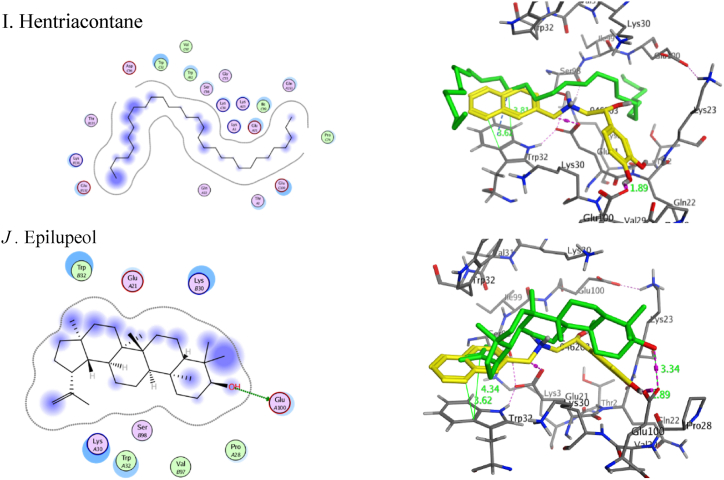
**2D (left) structure interaction poses of SOD1 with selected major identified compounds in the three investigated extracts and (right) visualization of intermolecular interactions co-crystallized ligand 946 with yellow color and the docked molecule with green color.** A, methyl hexadecanoate; B, methyl decanoate; C, methyl dodecanoate, D; Hexadecenoate, E; 4,8,12,16-tetramethyl heptadecan-4-olide; *F α*-tocospiro A; G*, D:C*-Friedo-*B':A′*-neogammacer-9(11)-ene, 3-methoxy-, (3β)-; H 6,10,14-trimethyl-2-pentadecanone; I, hentriacontane; J*, e*pilupeol.

We used the X-ray crystal structure of SOD1 bound to a naphthalene-catechol-linked molecule (PDB ID: 5YTO) to conduct molecular docking simulations in a computer model. To achieve a molecular docking study of methyl dodecanoate, hexadecenoate, methyl hexadecanoate, methyl decanoate, and 4,8,12,16-tetramethyl heptadecan-4-olide from *A.eggersii*, *D: C*-Friedo-*B':A′*-neogammacer-9(11)-ene, 3-methoxy-, (3β)- and *α*-tocospiro A from *C.macrospermum* and hentriacontane, epilupeol, and 6,10,14-trimethyl-2-pentadecanone from *J. caffra* engage the binding pocket at β-barrel loop II-strand 3 and overlay for the crystallized molecules (Fig. 5) with S score 0.61 to 1.52 Å and RMSD values of −8.3426 to −5.2633 kcal/mol. By redocking the co-crystallized ligand (PDB ID: 946) into SOD1 binding sites, the MOE molecular docking procedure was confirmed to be accurate. It binds and superimposes the crystallized molecules with RMSD values of 1.01 and an S score of −6.88 kcal/mol. The natural products impeded at the hydrophilic pocket lined with Thr2, Lys30, Lys23, Glu21, and Pro28 residues. Most displayed an H-bond with one of the polar residues (Table 4). The alkyl chain of the docked natural molecules extended to the hydrophobic pocket containing Trp32 moiety. It overlayed for the naphthalene ring, which is responsible for *π-π* stacking and inhibits Trp32 oxidation (Fig. 4).

**Fig. 6:**
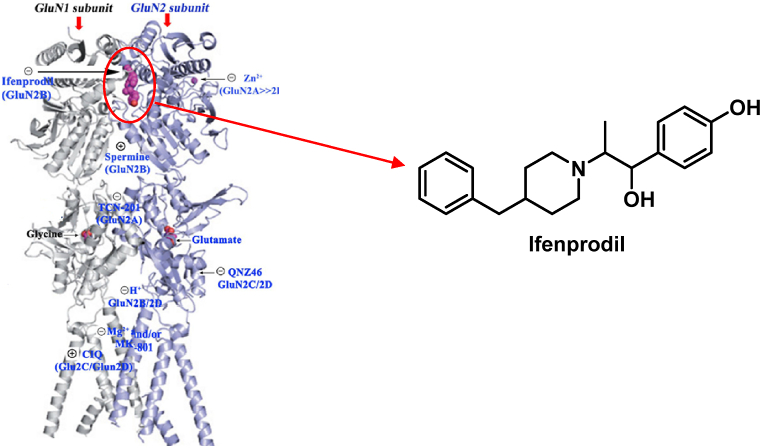
The crystal structure of the GluN1/GluN2 heterodimer reveals the presence of regulatory binding sites that can modulate the activity of the NMDAR (Paoletti P, 2013) and ifenprodil chemical structure.

NMDARs consist of tetrameric complexes with several homologous subunits (Fig. 6). The NMDAR subunits exhibit compositional flexibility, leading to a substantial diversity of receptor subtypes. It is crucial to determine if individual subtypes of receptors perform specific activities in the central nervous system (CNS) under both normal and pathological settings due to their varied biophysical, pharmacological, and signaling features [80]. Excitotoxicity is specifically triggered by NMDARs selective activation; that have NR2B subunits [81]. Hence, NR2B-NMDAR antagonists can potentially provide therapeutic advantages for various neurological disorders. Ifenprodil was the initial compound identified as an antagonist of the NR2B-NMDA receptor [82]. Regrettably, ifenprodil exhibits limited bioavailability in living organisms. This motivated scientific researchers to look for NR2B-NMDAR antagonists with unique binding mechanisms. Herein, we selected the crystal structure with the (PDB code: 5EWJ) for further calculations. The utilized protein structure consists of a dimer composed of GluN1 and GluN2B subunits of NMDAR amino-terminal domains (ATDs) (Fig. 6).

The ligand interacts within the dimer interface between the ATDs. Therefore, two chains (A and B) of the NMDAR were retained for the preparation. To verify the biological findings, the primary natural products from each extract were chosen for molecular docking analysis. This was done to understand better how these compounds interact inside the active region of NR2B-NMDARs. It binds the interdomain cavity between GluN1 and GluN2B subunits and superimposed for the crystallized molecules with RMSD values 0.419 to 1.23 Å and binding energy scores of −11.56 to −6.68 kcal/mol (Table 5).

**Fig. 7:**
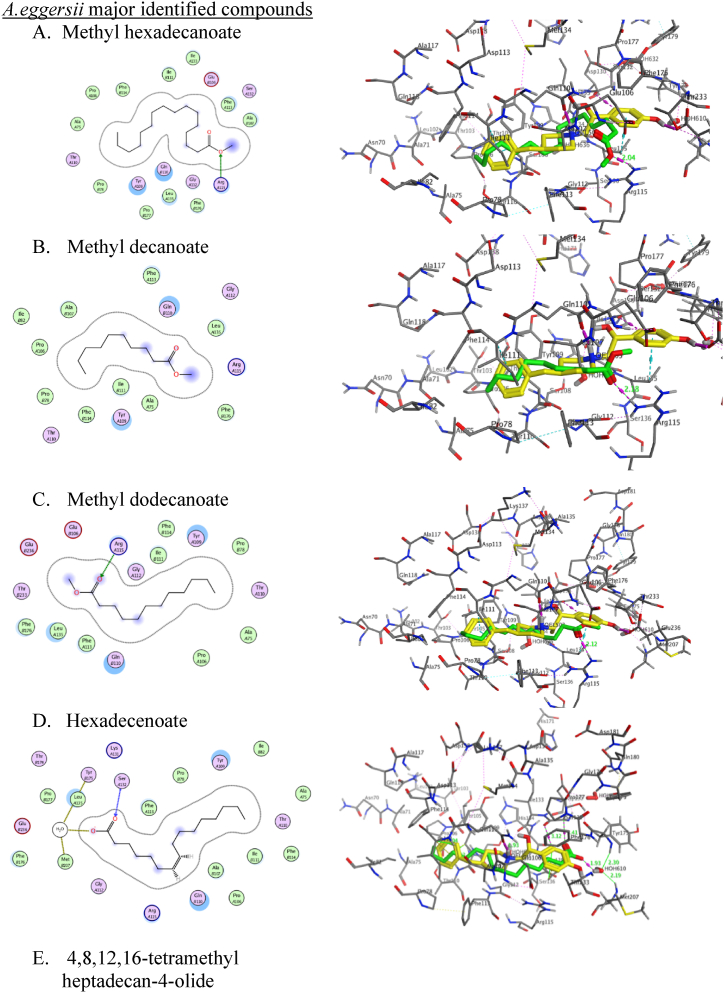

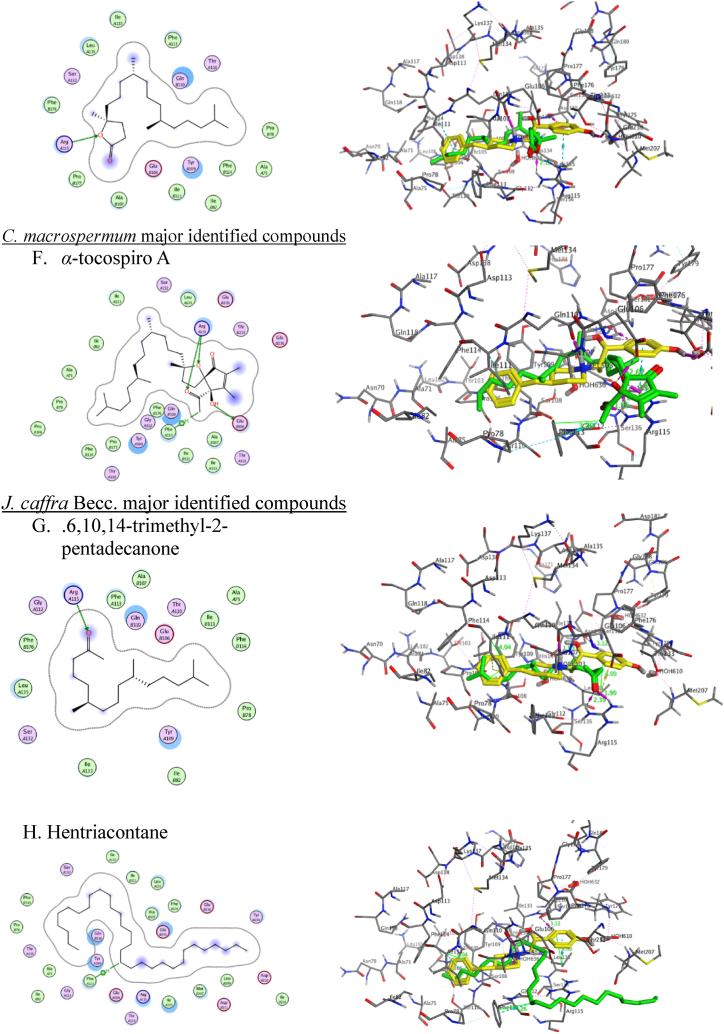
**2D (left) structure interaction poses of NR2B-NMDARs with selected major identified compounds in the three investigated extracts and (right) visualization of intermolecular interactions co-crystallized ligand QEL with yellow color and the docked molecule with green color**. A, methyl hexadecanoate; B, methyl decanoate; C, methyl dodecanoate; D, Hexadecenoate; E, 4,8,12,16-tetramethyl heptadecan-4-olide; F*, α*-tocospiroA; *G,* 6,10,14-trimethyl-2-pentadecanone; H, hentriacontane.

Herein, the co-crystallized ligand (PDB ID: QEL) was docked into NR2B-NMDARs binding sites to confirm the accuracy of the MOE molecular docking procedure. It binds and superimposes for the crystallized molecules with RMSD values of 1.0005 and S score −10.1571 kcal/mol. Examining the docking poses and orientation of the most potent compounds indicated that the left side pocket in the NMDA receptor has sufficient space to accommodate bulky groups. The aliphatic chain of the natural compounds extended to align with the benzyl hydrophobic moiety of ifenprodil and embedded into the hydrophobic pocket. This suggests that the site could potentially accommodate such compounds, leading to enhanced selectivity towards the NR2B subunit of the NMDA receptor. The hydrophobic pocket, consisting of Tyr109 and Leu135 on the GluN1b segment and Ile111, Phe176, and Pro177 on the GluN2B segment of the NMDA receptor, is a targetable pocket for drugs. Increasing the amount of carbon atoms in the drugs leads to an elevation in the RMSD value. The binding affinity of hentriacontane, methyl hexadecanoate, hexadecenoate, methyl dodecanoate, methyl decanoate are −11.5609, −8.2247, −7.9334, −7.3711 and −6.6813, respectively affected by descending the alkyl chain size. Moreover, the bulkiness of terminal alkyl chain enhance the RMSD value like the presence of isopropyl terminal group; *α*-tocospiro A, 4,8,12,16-tetramethyl heptadecan-4-olide and 6,10,14-trimethyl-2-pentadecanone leads to −9.3048, −8.2938 and −8.0331 kcal/mol, respectively. On the other hand, the rigidity and bulkiness of the terminal cyclic structure of *Epilupeol and D: C*-Friedo-*B':A′*-neogammacer-9(11)-ene, 3-methoxy-, (3β)- leads to decrease the binding affinity of active molecules to the hydrophobic pocket. Most natural compounds have terminal H-bond acceptors that lead to strong H-bond interactions at the right side of the binding pocket. The Arg115 observed H-bond with an oxygen atom of the ester group in Methyl hexadecanoate, Hexadecenoate, Methyl dodecanoate, and Methyl decanoate; an oxygen atom of the ketonic group in 6,10,14-trimethyl-2-pentadecanone; oxygen of 5-methyl dihydrofuran-2(3H)-one in 4,8,12,16-tetramethyl heptadecan-4-olide and bidentate H-bond with alpha-tocospiro A due to the presence of 9-acetyl-oxaspiro[4.4]non-7-en-6-one. Alpha-tocospiro A shows an extra H-bond with Glu106 and a carboxylate oxygen atom of hexadecenoate with Ser132. The docking results show that most of the natural compounds bind to NR2B-NMDARs and verify the credibility of the biological findings (Fig. 7). Although the docking study gave a deep insight into the critical structure features of the major bioactive metabolites, these compounds couldn't be subjected to structural modifications and optimization while coexisting in the total extract form. So, one of our future directions is to perform bio-guided isolation of the most active components and apply structural modifications to optimize the observed biological activity.

## 5 Conclusion

The lipophilic extracts of *A.eggersii* Burret, *C. macrospermum* H.Wendl. & Drude, and *J.caffra* Becc. (Family Arecaceae) showed a promising and comparable neuroprotective effect against glutamate-induced excitotoxicity. GC/MS identified the phytochemical profile of these extracts and revealed the presence of fatty acid derivatives, hydrocarbons (oxygenated and non-oxygenated), vitamins, and triterpenes. Accordingly, the neuroprotective effect is not due to a single component but to the unique blend of different bioactive phytochemicals. The identified constituents possess crucial lipophilic properties promoting them to cross the blood-brain barrier and reach the targeted sites for neuroprotection. The docking study showed the importance of alkyl hydrophobic moiety for binding to NR2B-NMDARs and SOD-1. Thus, decreasing the NMDAR-mediated neuro-excitotoxicity and fighting oxidative stress. Overall, this study provides the scientific basis for the possible pharmacological role of the investigated extracts as neuroprotective agents. Nevertheless, preclinical testing and appropriate dosage formulations are required.

## Studies in animals’ statement

The Faculty of Pharmacy's Ethical Animal Care and Use Committee approved the experimental protocol at Helwan University (approval number: 10A2023). It also complied with the NIH Guidelines for Animal Care (8th edition) and national guidelines for animal care (European Community Directive, 6/609/EEC).

## CRediT authorship contribution statement

**Fatma A. Moharram:** Writing – review & editing, Writing – original draft, Supervision, Resources, Methodology, Investigation, Formal analysis, Conceptualization. **Fadila M. Hamed:** Writing – original draft, Methodology, Data curation. **Elsayed K. El-Sayed:** Writing – review & editing, Writing – original draft, Methodology, Investigation, Formal analysis, Data curation. **Shimaa K. Mohamed:** Writing – review & editing, Writing – original draft, Methodology, Investigation, Formal analysis, Data curation. **Asmaa A. Ahmed:** Writing – review & editing, Writing – original draft, Methodology, Investigation, Formal analysis, Data curation. **Sabah H. Elgayed:** Writing – review & editing, Writing – original draft, Supervision, Resources, Methodology, Investigation, Formal analysis, Conceptualization. **Mohammed Abdelrazek:** Writing – review & editing, Writing – original draft, Methodology, Investigation, Formal analysis, Data curation. **Kuei-Hung Lai:** Writing – review & editing, Writing – original draft, Resources, Funding acquisition, Formal analysis. **Yara E. Mansour:** Writing – review & editing, Writing – original draft, Formal analysis, Data curation. **Mohamed S. Mady:** Writing – review & editing, Writing – original draft, Supervision, Resources, Methodology, Investigation, Formal analysis, Conceptualization. **Heba E. Elsayed:** Writing – review & editing, Writing – original draft, Supervision, Resources, Methodology, Investigation, Formal analysis, Conceptualization.

## Data and code availability statement

Data will be made available on request.

## Funding

The grants that supported this work were from the 10.13039/100020595National Science and Technology Council of Taiwan (MOST 111-2321-B-255-001 and MOST 111-2320-B-038-040-MY3).

## Declaration of competing interest

The authors declare the following financial interests/personal relationships which may be considered as potential competing interests: The grants that supported this work were from the 10.13039/100020595National Science and Technology Council of Taiwan (MOST 111-2321-B-255-001 and MOST 111-2320-B-038-040-MY3).

## List of abbreviations

ALS: Automatic liquid sampler
BBB: Blood-brain barrier
ER: Endoplasmic reticulum
GC/MS: Gas chromatography/Mass spectrometry
GSH: Reduced glutathione
iNOS: Induced nitric oxide synthetase
MC/MSD: Gas Chromatograph/Mass Selective Detector
MDA: Malondialdehyde
MSG: Monosodium glutamate
NF-κB: Nuclear factor kappa-b
NMDAR: N-methyl-D-aspartate receptor
NO: Nitric oxide
8-OHdG: 8-Hyroxydeoxyguanosine
PEE: Petroleum ether extract
RNS: Reactive nitrogen species
ROS: Reactive oxygen species
SOD: Superoxide dismutase

## Footnotes

Appendix A Supplementary data to this article can be found online at https://doi.org/10.1016/j.heliyon.2024.e39207.
