# Supplementary material for: Chemical characterization, neuroprotective effect, and in-silico evaluation of the petroleum ether extract of three palm tree species against glutamate-induced excitotoxicity in rats
Source: Heliyon. 2024 Oct 11;10(20):e39207. doi: 10.1016/j.heliyon.2024.e39207 (PMC11620252; doi:10.1016/j.heliyon.2024.e39207)
Supplement: Multimedia component 1 [file mmc1.docx]

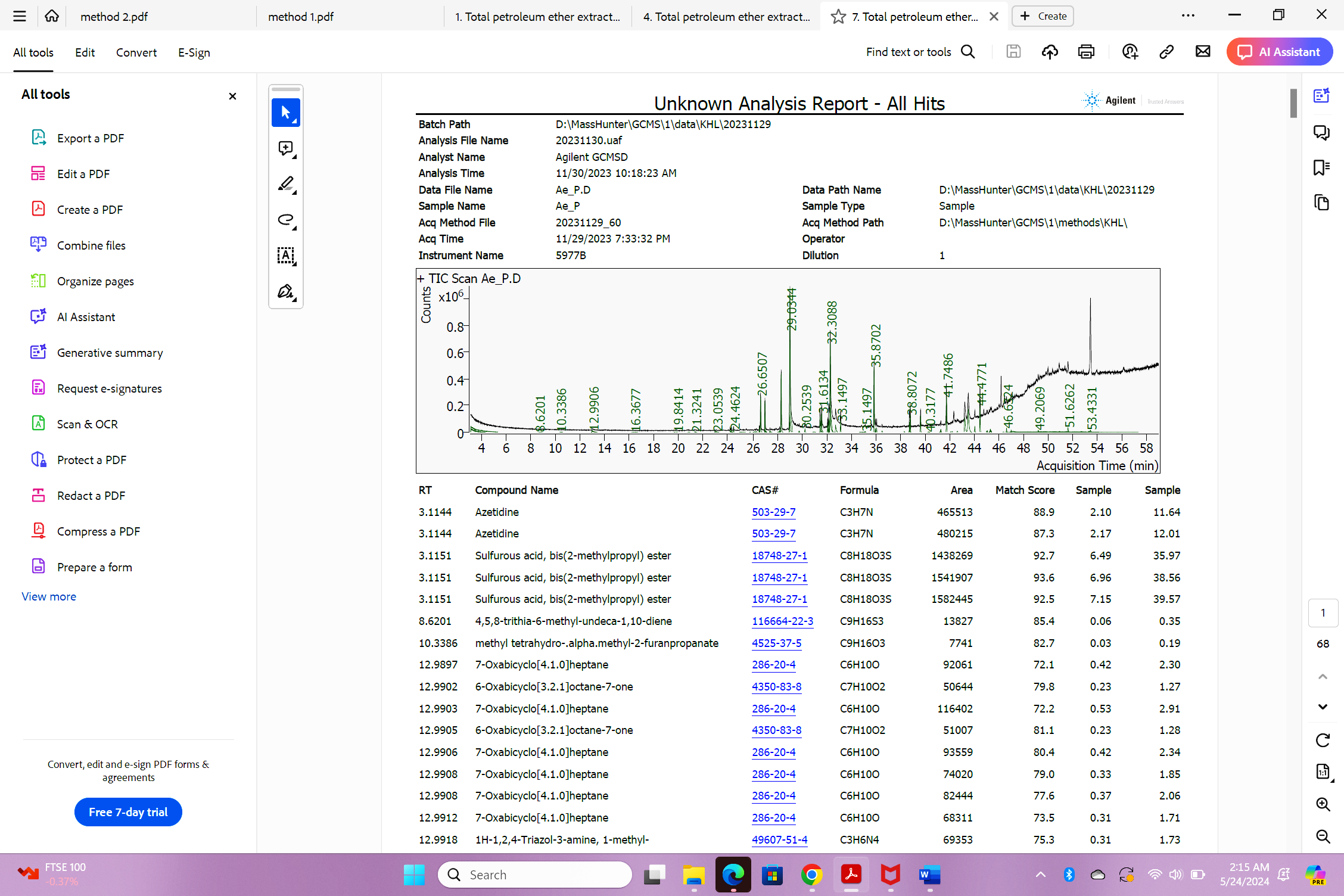


**Figure S1: TIC of the petroleum ether extract of *Aiphanes eggersii* Burret leaves**


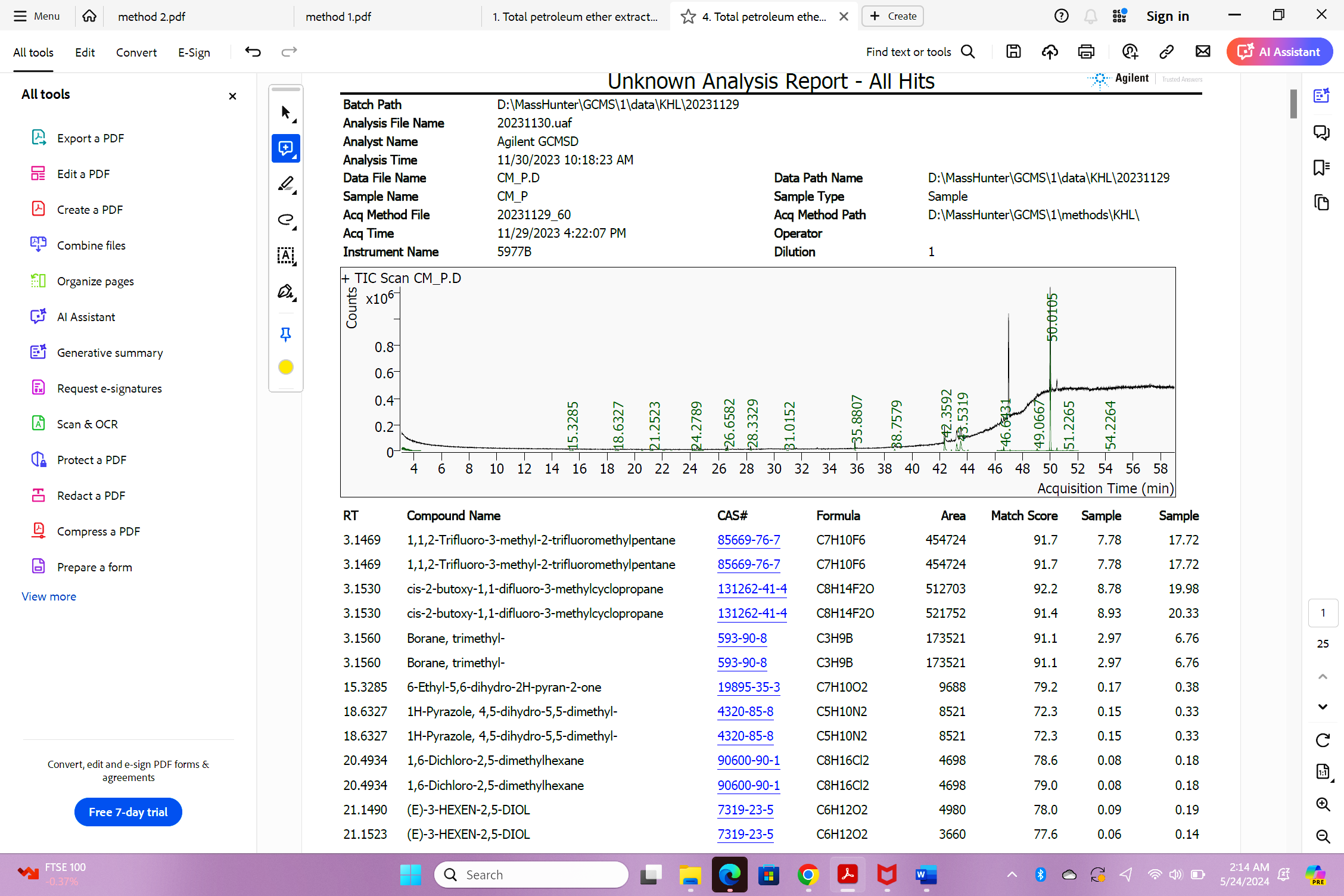


**Figure S2: TIC of the petroleum ether extract of *Carpoxylon macrospermum* H.Wendl. & Drude leaves**


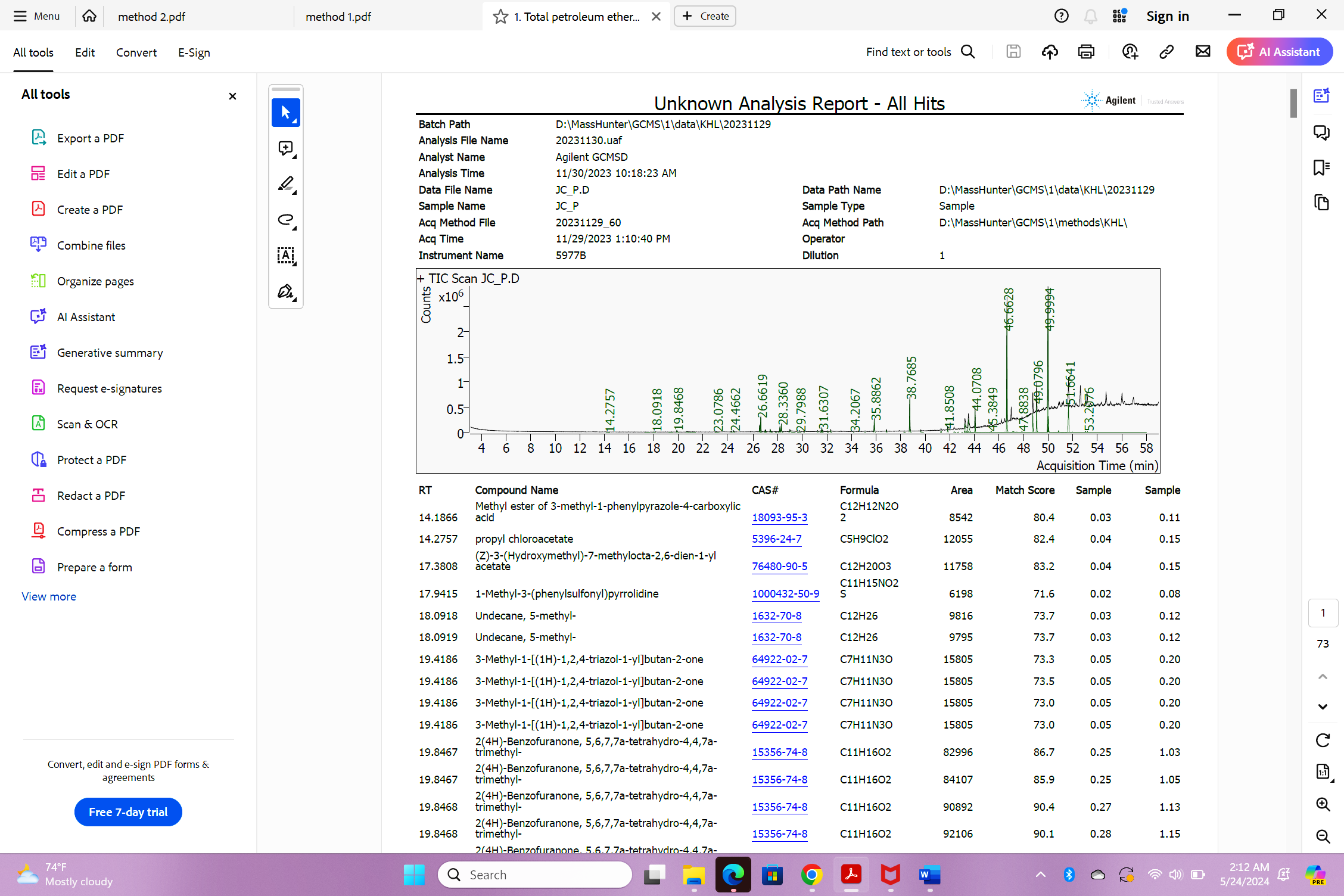


**Figure S3: TIC of the petroleum ether extract of *Jubaeopsis caffra* Becc. leaves**

**References list for Tables (1-3)**

[1s] Ding, L.-F., Peng, L.-Y., Zhou, H.-F., Song, L.-D., Wu, X.-D., Zhao, Q.-S., 2020. Artemilavanolides A and B, two sesquiterpenoids with a 6-oxabicyclo[3.2.1]octane scaffold from Artemisia lavandulaefolia. Tetrahedron Letters 61(21), 151872. <https://doi.org/10.1016/j.tetlet.2020.151872>.

[2s] Hamed, A.R., Abou Zeid, A.H., El-Rafie, H., Kandil, Z.A., El-Akad, R.H., Farag, M., 2020. Bioactivity Guided Investigation of Caryota mitis &amp; Caryota urens Chemopreventive Activity via In Vitro and In Silico Studies. Egyptian Journal of Chemistry 63(12), 5071-5086. 10.21608/ejchem.2020.30737.2655.

[3s] Francke, W., Schulz, S., 2010. 4.04 - Pheromones of Terrestrial Invertebrates, in: Liu, H.-W., Mander, L. (Eds.), Comprehensive Natural Products II. Elsevier, Oxford, pp. 153-223.

[4s] Vanitha, V., Vijayakumar, S., Nilavukkarasi, M., Punitha, V.N., Vidhya, E., P.K, P., 2020. Heneicosane—A novel microbicidal bioactive alkane identified from Plumbago zeylanica L. Industrial Crops and Products 154, 112748. 10.1016/j.indcrop.2020.112748.

[5s] Rawat, A., Kholiya, S., Chauhan, A., Venkatesha, K.T., Kumar, D., Upadhyay, R.K., Padalia, R.C., 2023. Chemical variability on Zingiber zerumbet (L.) Roscoe ex Sm. essential oil with respect to different comminution methods. Biochemical Systematics and Ecology 106, 104574. https://doi.org/10.1016/j.bse.2022.104574.

[6s] Inagaki, S., Fujikawa, S., Wada, Y., Kumazawa, K., 2015. Identification of the possible new odor-active compounds "12-methyltridecanal and its analogs" responsible for the characteristic aroma of ripe Gouda-type cheese. Biosci Biotechnol Biochem. 5(1347-6947 (Electronic)), 2098.

[7s] Kaur, S., Sharma, P., Bains, A., Chawla, P., Sridhar, K., Sharma, M., Inbaraj, B.S., 2022. Antimicrobial and Anti-Inflammatory Activity of Low-Energy Assisted Nanohydrogel of Azadirachta indica Oil, Gels.

[8s] Al-Qudah, M.A., 2013. Chemical composition of essential oil from Jordanian Lupinus varius L. Arabian Journal of Chemistry 6(2), 225-227. https://doi.org/10.1016/j.arabjc.2011.01.012.

[9s] AbouZeid, E., Hussein, R., Afifi, A., Salama, A., Youssef, F., El-Ahmady, S., Ammar, N., 2022. Phytochemical Profile of Phoenix rupicola T. Anderson Seeds and In Vitro Evaluation of their Estrogenic Activity using MCF-7 Cell Lines. Egyptian Journal of Chemistry 65(132), 1457-1464. 10.21608/ejchem.2022.129433.5731.

[10s] Lim, D.K., Mo, C., Lee, D.-K., Long, N.P., Lim, J., Kwon, S.W., 2018. Non-destructive profiling of volatile organic compounds using HS-SPME/GC–MS and its application for the geographical discrimination of white rice. Journal of Food and Drug Analysis 26(1), 260-267. <https://doi.org/10.1016/j.jfda.2017.04.005>.

[11s] Maher, S., Geoghegan, C., Brayden, D.J., 2023. Safety of surfactant excipients in oral drug formulations. Advanced Drug Delivery Reviews 202, 115086. <https://doi.org/10.1016/j.addr.2023.115086>.

[12s] Ding, D., Zhu, M., Huang, Z., Song, Z., 1989. Headspace Analysis of Osmanthus fragrans Lour. Journal of Essential Oil Research 1(6), 295-297. 10.1080/10412905.1989.9697802.

[13s] Tyagi, S., Tripathi, R., 2023. Hexadecanoic acid methyl ester, a potent hepatoprotective compound in leaves of Pistia stratiotes L

[14s] Srinivasan, K., Altemimi, A.B., Narayanaswamy, R., Vasantha Srinivasan, P., Najm, M.A.A., Mahna, N., 2023. GC-MS, alpha-amylase, and alpha-glucosidase inhibition and molecular docking analysis of selected phytoconstituents of small wild date palm fruit (Phoenix pusilla). Food Science & Nutrition 11(9), 5304-5317. https://doi.org/10.1002/fsn3.3489

[15s] Hpoo, M.K., Mishyna, M., Prokhorov, V., Arie, T., Takano, A., Oikawa, Y., Fujii, Y., 2020. Potential of Octanol and Octanal from Heracleum sosnowskyi Fruits for the Control of Fusarium oxysporum f. sp. lycopersici, Sustainability.

[16s] Hameed, I., Sosa, A., Bagi, S., 2016. Analysis of bioactive chemical compounds of Euphorbia lathyrus using gas chromatography-mass spectrometry and Fourier-transform infrared spectroscopy. Journal of Pharmacognosy and Phytotherapy 8, 109-126. 10.5897/JPP2015.0371.

[17s] Abdelrahim, M.S., Abdel-Baky, A.M., Backheet, E.Y., Bayoumi, S.A.L., 2022. Botanical study and fatty acids characterization of the leaves of Dypsis pembana (h.e. moore) beentje &amp;j. dransf. family Arecaceae cultivated in egypt. Bulletin of Pharmaceutical Sciences Assiut University 45(1), 99-118. 10.21608/bfsa.2022.239248.

[18s] Lahlou, A., Chileh-Chelh, T., Lyashenko, S., Rincón-Cervera, M.Á., Rodríguez-García, I., López-Ruiz, R., Urrestarazu, M., Guil-Guerrero, J.L., 2022. Arecaceae fruits: Fatty acids, phenolic compounds and in vitro antitumor activity. Food Bioscience 50, 102181. https://doi.org/10.1016/j.fbio.2022.102181.

[19s] Rodríguez-Leyes, E., Canavaciolo, V., Marrero Delange, D., Enríquez, A., Fajardo, Y., 2007. Fatty Acid Composition and Oil Yield in Fruits of Five Arecaceae Species Grown in Cuba. JAOCS, Journal of the American Oil Chemists' Society 84, 765-767. 10.1007/s11746-007-1103-z.

[20s] Ojha, D., Das, R., Sobia, P., Dwivedi, V.P., Ghosh, S., Samanta, A., Debprasad, C., 2015. Pedilanthus tithymaloides Inhibits HSV Infection by Modulating NF-κB Signaling. PloS one 10, e0139338. 10.1371/journal.pone.0139338.

[21s] Pucot, J., Dapar, M.L., Demayo, C., 2021. Qualitative Analysis of the Antimicrobial, Phytochemical and GC-MS profile of the Stem ethanolic extract from Anodendron borneense (King and Gamble). Journal of Complementary Medicine Research 12, 231. 10.5455/jcmr2021.12.02.27.

[22s] Okechukwu, P.N., 2020. Evaluation of anti-inflammatory, analgesic, antipyretic effect of eicosane, pentadecane, octacosane, and heneicosane. Asian Journal of Pharmaceutical and Clinical Research 13(4), 29-35. 10.22159/ajpcr.2020.v13i4.36196.

[23s] Salhi, S., Chentouf, M., Harrak, H., Rahim, A., Çakir, C., Çam, D., Öztürk, M., Hamidallah, N., Cabaraux, J.-F., El Amiri, B., 2023. Assessment of physicochemical parameters, bioactive compounds, biological activities, and nutritional value of the most two commercialized pollen types of date palm (Phoenix dactylifera L.) in Morocco. Food Science and Technology International, 10820132231168914. 10.1177/10820132231168914.

[24s] Xu, Y., Liang, B., Kong, C., Sun, Z.A.-O., 2021. Traditional Medicinal Plants as a Source of Antituberculosis Drugs: A System Review. BioMed research international, 9910365.

[25s] El-Hawary, S.S., Owis, A.I., Abo El-Ela, S.O., Elwekeel, A., 2022. Nutritional Evaluation and GC/MS Analysis of Lipophilic Fractions of Livistona australis Leaves and Fruits. Egyptian Journal of Chemistry 65(5), 291-295. 10.21608/ejchem.2021.98793.4625.

[26s] Lykholat, Y., Khromykh, N., Didur, O., Okovytyy, S., Sklyar, T., Davydov, V., Lykholat, T., Kovalenko, I., 2021. Soluble cuticular wax composition and antimicrobial activity of the fruits of Chaenomeles species and an interspecific hybrid. Biosystems Diversity 29(4), 334-339.

[27s] Cupido, M., De-Nova, A., Guerrero-González, M.L., Pérez-Vázquez, F.J., Méndez-Rodríguez, K.B., Delgado-Sánchez, P., 2022. GC-MS analysis of phytochemical compounds of Opuntia megarrhiza (Cactaceae), an endangered plant of Mexico. PeerJ Organic Chemistry 4, e5.

[28s] Knapp, H., Straubinger, M., Fornari, S., Oka, N., Watanabe, N., Winterhalter, P., 1998. (s)-3, 7-dimethyl-5-octene-1, 7-diol and related oxygenated monoterpenoids from petals of Rosa damascena Mill. Journal of Agricultural Food Chemistry 46(5), 1966-1970.

[29s] Jadhav, V., 2018. GC-MS Screening of Some Bioactive Compounds from Methanolic Extract of Medicinally Relevant Wild Edible Plant Parts. International Journal of Scientific Research in Science and Technology 4, 49-56.

[30s] Junairiah, Irmayanti, N.D., Nurhariyati, T., 2020. Bioactive compounds profile and antimicrobe activities of n-hexane and ethyl acetate extracts of Piper retrofractum fruit. CABI Databases 36, 329-332

[31s] Esquivel-Ferriño, P.C., Favela-Hernández, J.M.J., Garza-González, E., Waksman, N., Ríos, M.Y., Camacho-Corona, M.d.R., 2012. Antimycobacterial activity of constituents from Foeniculum vulgare var. dulce grown in Mexico. Molecules 17(7), 8471-8482.

[32s] Chen, J., Gonzalez, R., 2023. Engineering Escherichia coli for selective 1-decanol production using the reverse β-oxidation (rBOX) pathway. Metabolic Engineering 79, 173-181. <https://doi.org/10.1016/j.ymben.2023.07.006>.

[33s] Ayubova, M., Guelleh, Z.O., Guelleh, M.O., Brévard, H., Baldovini, N., 2019. Analytical investigations on Boswellia occulta essential oils. Phytochemistry 164, 78-85. <https://doi.org/10.1016/j.phytochem.2019.04.020>.

[34s] Kawakami, M., Kobayashi, A., 1991. Volatile constituents of green mate and roasted mate. Journal of Agricultural Food Chemistry 39(7), 1275-1279.

[35s] El Hawary, S., Ibrahim, N.A., Aly, H.F., Matlob, A., Dosoky, M.M., Abd Elhady Deabes, D., Selim, N.M., 2021. Comparative phytochemical and biological studies of lipoidal matter of Ipomoea tricolor (Cav.) and Ipomoea fistulosa (Mart. Ex Choisy) growing in Egypt. Egyptian Journal of Chemistry 64(4), 1845-1857.

[36s] Hardell, H.-L., Nilvebrant, N.-O., 1999. A rapid method to discriminate between free and esterified fatty acids by pyrolytic methylation using tetramethylammonium acetate or hydroxide. Journal of Analytical and Applied Pyrolysis 52(1), 1-14. <https://doi.org/10.1016/S0165-2370(99)00035-2>.

[37s] Prastiyanto, M.E., Solekha, R., Rohmah, L.A., Rachmawati, Y., Aini, N.S.J.G.J.o.B., 2023. 3, 3-dimethyl-octane from Physalis peruviana as promising anti-DENV via ADMET prediction of pkCSM open webserver. 3(1), 23-30.

[38s] Govindarajan, N., Cheekala, U., Arcot, S., Sundharamoorthy, S., Duraisamy, R., Raju, I., 2016. GC-MS Analysis of n-hexane Extract of Stem Bark of Symplocos crataegoides Buch.-Ham. ex D. Don. Pharmacognosy Journal 8, 520-524. 10.5530/pj.2016.6.2.

[39s] Dash, S., Bohidar, J., Das, C., Mohanty, A., Meher, A., Hota, R., 2023. Evaluation of Anthelmintic Activity and GC-MS Characterization of Urochloa distachya (L.). International Journal of Pharmaceutical Investigation 13(2).

[40s] Hamidi, N., Ziane, L., Djellouli, M., Lazouni, H., 2016. Chemical characterization by GC-MS from the aerial parts of Fagonia longispina (Zygophyllaceae). Asian J Pharm Clin Res 9(1), 175-176.

[41s] Xu, H., Lv, L., Hu, S., Song, D., 2010. High-performance liquid chromatographic determination of hexanal and heptanal in human blood by ultrasound-assisted headspace liquid-phase microextraction with in-drop derivatization. Journal of Chromatography 1217(16), 2371-2375.

[42s] Frunzete, M.-E., Rodideal, T., Grigore, M.-N., Bădulescu, L., Ciocan, R.M., Zamfirache, M.-M., 2023. Investigations on the chemical composition of volatile oils extracted from the leaves of spontaneous and cultivated Taxus baccata L. trees. Notulae Botanicae Horti Agrobotanici Cluj-Napoca 51(4), 13383-13383.

[43s] Kaushalya, K., Kumara, A., Ranaweera, G., Rathnayaka, R., Silwa, M., Mubarak, A., 2021. Host plant volatiles released by Bracharaira brizantha and Desmodium spp. and their effects on the behaviour of fall armyworm.

[44s] Elmadni, H., Mishyna, M., Fujii, Y., 2019. Identification of 1-decyne as a new volatile allelochemical in baobab (Adansonia digitata) from Sudan. Afr. J. Agric. Res 14(21), 907-914.

[45s] Findlay, J.A., Yayli, N., Calhoun, L.A., 1991. Novel sulfated hydrocarbons from the sea cucumber Cucumaria frondosa. Journal of natural products 54(1), 302-304.

[46s] Valentin, H., Schönebaum, A., Steinbüchel, A.J.A.m., biotechnology, 1996. Identification of 5-hydroxyhexanoic acid, 4-hydroxyheptanoic acid and 4-hydroxyoctanoic acid as new constituents of bacterial polyhydroxyalkanoic acids. 46, 261-267.

[47s] Tariq, M., Ali, S., Ahmad, F., Ahmad, M., Zafar, M., Khalid, N., Khan, M.A.J.F.P.T., 2011. Identification, FT-IR, NMR (1H and 13C) and GC/MS studies of fatty acid methyl esters in biodiesel from rocket seed oil. 92(3), 336-341.

[48s] Katiyar, D., Singh, R.K., Singh, S., Singh, D., Singh, V., 2011. Isolation and characterization of n-docosane from heartwood of Berberis aristata. International Journal of Pharmaceutical Sciences Research 2(2), 331.

[49s] Aja, P., Nwachukwu, N., Ibiam, U., Igwenyi, I., Offor, C., Orji, U.J., 2014. Chemical constituents of Moringa oleifera leaves and seeds from Abakaliki, Nigeria. American Journal of Phytomedicine Clinical Therapeutics 2(3), 310-321.

[50s] Choi, H.-S.J.T.K.J.o.F., Nutrition, 2021. Analysis of Essential Oils Extracted from Fresh and Shade-dried Leaves of Synurus deltoides (Arr.) Nakai. 34(2), 224-232.

[51s] Zhou, Y., Wu, J., Lin, S., He, J., Deng, Y., He, J., Cheng, D.J.P.M.S., 2022. The synergistic effects of rosehip oil and matrine against Icerya aegyptiaca (Douglas)(Hemiptera: Coccoidea) and the underlying mechanisms. 78(8), 3424-3432.

[52s] Ilango, S., Jayachandran, P., Sivaswamy, A., Subaramaniyam, U., Sukumar, S., Nirmaladevi, R., 2022. Characterization and Anticancer Activity of Annona Muricata Leaf Fractions against T-Cell Acute Lymphoblastic Leukemia Cell Line (Molt-3). inflammation 13, 14.

[53s] Babu, Y.R., Satapathy, P., Krishna, C.M., Ramalakshmana, J., Padal, S., 2023. GC-MS analysis and screening of antimicrobial potentialities of the medicinal herb Peperomia pellucida (L.) Kunth. Journal of Xidian University 1376-1389.

[54s] Rajalakshmi, R., Vaidehi, J., Krishnappa, K., 2022. The naturally available phyto-products of Indian medicinal plants against adulticidal activity of human vector mosquitoes. CABI Databases 43, 80-87.

[55s] Save, S., 2015. Determination of 1, 2-Benzenedicarboxylic acid, bis (2-ethylhexyl) ester from the twigs of Thevetia Peruviana as a Colwell Biomarker. Journal of Innovations in Pharmaceuticals and Biological Sciences 2, 349-362.

[56s] Tessmann, D., Dianese, J., 2002. Hentriacontane: a leaf hydrocarbon from Syzygium jambos with stimulatory effects on the germination of urediniospores of Puccinia psidii. Fitopatologia Brasileira 27. 10.1590/S0100-41582002000500017.

[57s] Siles, L., Cela, J., Munné-Bosch, S., 2013. Vitamin E analyses in seeds reveal a dominant presence of tocotrienols over tocopherols in the Arecaceae family. Phytochemistry 95, 207-214. https://doi.org/10.1016/j.phytochem.2013.07.008.

[58s] Gnanashree, G., Sirajudeen, P.M., 2018. Determination of bioactive compounds in ethanolicextract of Caralluma indica using GC-MS technique. J Journal of Pharmacognosy and Phytochemistry 7(6), 1675-1677.

[59s] Yang, K., Yang, Y., Wu, X., Zheng, F., Xu, G., Yang, S., Jin, G., Clements, D.R., Shen, S., Zhang, F., 2024. Allelopathic Potential and Chemical Composition of Essential Oil from the Invasive Plant Acmella radicans, Agronomy.

[60s] Mohammed, M.H.H., Fouad, M.A., 2022. Chemical and biological review on various classes of secondary metabolites and biological activities of Arecaceae (2021-2006). Journal of advanced Biomedical and Pharmaceutical Sciences 5(3), 113-150. 10.21608/jabps.2022.126338.1149.

[61s] Hussien, T.A., El-Amir, D.A., Radwan, U.A.A., El-Sayed, M.A., Mohamed, A.E.-H.H., El-Damarany, H., 2023. Antimicrobial Benzofuran Derivatives and Chemosystematic Significance of Senecio glaucus L. (Asteraceae). Egyptian Journal of Chemistry 66(10), 575-583. 10.21608/ejchem.2023.168069.7076.

[62s] Park, J.S., Rehman, I.U., Choe, K., Ahmad, R.A.-O., Lee, H.J., Kim, M.O., 2023. A Triterpenoid Lupeol as an Antioxidant and Anti-Neuroinflammatory Agent: Impacts on Oxidative Stress in Alzheimer's Disease. LID - 10.3390/nu15133059 [doi] LID - 3059. Nutrients 15(2072-6643 (Electronic)), 3059.
